# A protein complex of LCN2, LOXL2 and MMP9 facilitates tumour metastasis in oesophageal cancer

**DOI:** 10.1002/1878-0261.13529

**Published:** 2023-10-04

**Authors:** Qiaoxi Xia, Zepeng Du, Mantong Chen, Xiao Zhou, Wenjing Bai, Xiaoqi Zheng, Ling Lin, Yan Zhao, Jiyu Ding, Zhisheng Wu, Haiying Zou, Shaohong Wang, Liyan Xu, Enmin Li, Bingli Wu

**Affiliations:** ^1^ Department of Biochemistry and Molecular Biology Shantou University Medical College China; ^2^ Central Laboratory Shantou Central Hospital China; ^3^ Department of Pathology Shantou Central Hospital China; ^4^ Institute of Oncologic Pathology Shantou University Medical College China

**Keywords:** extracellular matrix, invasion, migration, protein ternary complex

## Abstract

During malignant tumour development, the extracellular matrix (ECM) is usually abnormally regulated. Dysregulated expression of lysyl oxidase‐like 2 (*LOXL2*), matrix metalloproteinase 9 (*MMP9*) and lipocalin 2 (*LCN2*) are associated with ECM remodelling. In this study, protein–protein interaction assays indicated that LCN2 and LOXL2 interactions and LCN2 and MMP9 interactions occurred both intracellularly and extracellularly, but interactions between LOXL2 and MMP9 only occurred intracellularly. The LCN2/LOXL2/MMP9 ternary complex promoted migration and invasion of oesophageal squamous cell carcinoma (ESCC) cells, as well as tumour growth and malignant progression *in vivo*, while the iron chelator deferoxamine mesylate (DFOM) inhibited ESCC tumour growth. Co‐overexpression of *LCN2*, *LOXL2* and *MMP9* enhanced the ability of tumour cells to degrade fibronectin and Matrigel, increased the formation and extension of filopodia, and promoted the rearrangement of microfilaments through upregulation of *profilin 1*. In addition, the LCN2/LOXL2/MMP9 ternary complex promoted the expression of *testican‐1* (*SPOCK1*), and abnormally activated the FAK/AKT/GSK3β signalling pathway. In summary, the LCN2/LOXL2/MMP9 ternary complex promoted the migration and invasion of cancer cells and malignant tumour progression through multiple mechanisms and could be a potential therapeutic target.

## Graphical abstract

The LCN2/LOXL2/MMP9 complex remodels the ECM and activates the FAK/AKT/GSK3β signalling pathway to enhance *SPOCK1* expression, promoting the migration and invasion of oesophageal cancer cells. The degradation of ECM is inhibited after treatment with the DFOM targeting the LCN2 and PI3K inhibitor, wortmannin. Therefore, LCN2 and PI3K(S473) are potential therapeutic targets for oesophageal cancer.

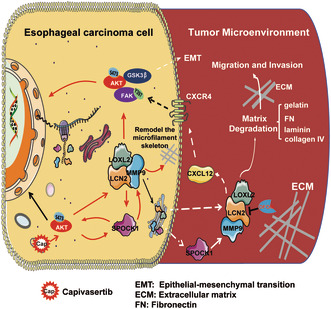

**Abbreviations**
DFOM: deferoxamine mesylate
ECM: extracellular matrix
EMT: epithelial‐mesenchymal transformation
ESCC: oesophageal squamous cell carcinoma
LCN2: lipocalin 2
LOXL2: lysyl oxidase‐like 2
MMP9: matrix metalloproteinase 9
MMPs: matrix metalloproteinases
NGAL: neutrophil gelatinase‐associated lipocalin

## 1 Introduction

Oesophageal cancer currently ranks seventh in terms of incidence (604 000 new cases) and sixth in mortality overall (544 000 deaths), the latter signifying that oesophageal cancer was responsible for 1 in every 18 cancer deaths in 2020 [1]. Over the past four decades, the global incidence of oesophageal cancer has increased significantly [2], with oesophageal squamous cell carcinoma (ESCC) being the most common histologic subtype worldwide and China being one of the countries with a high incidence of ESCC [3]. Oesophageal carcinoma cannot be easily diagnosed at the early stages, which leads to most patients being diagnosed at an advanced stage, thus losing the opportunity for early treatment. This problem is exacerbated because the malignant pathogenesis of oesophageal carcinoma remains not fully understood.

Tumour progression is strongly linked with the tumour microenvironment. The extracellular matrix (ECM) is the main component of the microenvironment, and production, secretion, degradation and rearrangement of the ECM are strictly regulated [4]. However, homeostasis of the ECM is dynamically disrupted during cancer progression. Matrix metalloproteinases (MMPs), especially *MMP9*, are thought to be particularly important for cancer invasion and metastasis, due to their ability to degrade ECM barriers. *MMP9* is involved in multiple biological processes, such as proteolytic degradation of the ECM, alteration of cell–cell and cell‐ECM interactions, cleavage of cell surface proteins, and cleavage of proteins in the extracellular environment [5].

*LCN2*, also known as neutrophil gelatinase‐associated lipocalin (*NGAL*), is a secreted glycoprotein, and its abnormal expression in various human solid tumours plays a vital role in the epithelial–mesenchymal transformation (EMT), angiogenesis, and cell migration and invasion [6, 7]. LCN2 and MMP9 form a protein complex to protect MMP9 from protein degradation, thereby prolonging MMP9 enzymatic activity [8]. The interaction of LCN2 with MMP9 plays a crucial role in modulating the metastatic phenotype of cancer cells, and this interaction correlates with the aggressive behaviour of neoplastic cells in several types of cancer [9]. In our previous study, we found *LCN2* to be highly expressed in patients with ESCC and associated with a poor prognosis [10]. We also demonstrated that LCN2 promoted the migration and invasion of ESCC cells to increase MMP9 activity through a novel positive feedback loop [11].

*LOXL2* (lysyl oxidase‐like 2 protein), a member of the lysyl oxidase family, is a secreted copper‐dependent amine oxidase. One important role of LOXL2 is to catalyse the covalent cross‐linking of collagen and elastin in the ECM, which contributes to the strength of collagen fibrils and elasticity of elastic fibres [12]. We have found that high expression of *LOXL2* is associated with lymph node metastasis and a poor prognosis in ESCC and that LOXL2 induces cytoskeletal reorganization and ezrin phosphorylation, which subsequently promotes tumour cell invasion and metastasis in ESCC [13, 14]. Accumulating evidence suggests that LOXL2 promotes invasion and metastasis, angiogenesis and malignant transformation in many solid tumours, and aberrant high expression of *LOXL2* is usually correlated with a poor prognosis [15].

Taken together, *LCN2*, *LOXL2*, and *MMP9* are highly expressed in ESCC, and are emerging as promising therapeutic targets in many types of cancer [9, 15, 16]. In addition to their individual diverse functions, LCN2, MMP9 and LOXL2 are all secreted proteins and are involved in reshaping the ECM, contributing to the progression, invasion, and migration of tumours. Therefore, we investigated whether LCN2, LOXL2, and MMP9 are interrelated in ECM remodelling, and whether LCN2/LOXL2/MMP9 form a complex to play a synergistic role in tumour progression.

## 2 Materials and methods

### 2.1 Cells and cell culture

ESCC cell lines KYSE140 (RRID: CVCL_1347), KYSE150 (RRID: CVCL_1348), KYSE180 (RRID: CVCL_1349), KYSE30 (RRID: CVCL_1351), KYSE410 (RRID: CVCL_1352), KYSE450 (RRID: CVCL_1353), KYSE510 (RRID: CVCL_1354) (established by Dr. Shimada Yutaka, Faculty of Medicine, Kyoto University, Japan), and TE3 (established by Dr. Nishihira, Tohoku University School of Medicine, Japan) were used for this study. The sources and STR genotyping of these cell lines have been described previously [11, 14]. ESCC cells and human embryonic kidney 293T cells were grown in RPMI‐1640 medium (HyClone, Logan, UT, USA) supplemented with 10% heat‐inactivated foetal bovine serum (Life Technologies, Gaithersburg, MD, USA) and 1% penicillin–streptomycin (Meijin, Guangzhou, China). Cells (free of mycoplasma contamination) were cultured in a 5% CO_2_ and 37 °C cell culture incubator and passaged by 0.25% trypsin digestion.

### 2.2 Plasmid preparation

Expression plasmids, including pcDNA3.1‐LCN2, pcDNA3.1‐LCN2‐HA, pCMV3‐MMP9‐Flag, pcDNA3.1‐LOXL2‐HA, pcDNA3.1‐LOXL2‐Flag, ΔLOXL2(545‐774aa)‐HA, ΔLOXL2(1‐544aa)‐HA, were described in our previous studies [11, 17]. pCMV3‐MMP9‐Flag was purchased from Sino Biological (Beijing, China). Plasmids were maintained in Trans5α *Escherichia coli* (CD201, TransGen, Beijing, China), and were harvested using an EZgene™ EndoFree plasmid miniprep kit (PD1212‐01, BIOMIGA, San Diego, CA, USA). Plasmids were transfected with Lipofectamine 3000 (L3000‐015, Thermo, Waltham, MA, USA) according to the manufacturer's instructions. A cell line stably overexpressing *LCN2*/*LOXL2*/*MMP9* was obtained by transfection using a Neon™ Transfection System (MPK5000S, Thermo), with parameter settings of 1400 V, 20‐ms pulse width, and two pulses, followed by selection in hygromycin B (10687010, Thermo) and Geneticin™ Selective Antibiotic (G418 Sulfate, 10131027, Thermo) for 2 weeks.

### 2.3 Protein extraction and concentration of conditioned medium

Cells were seeded in a 10‐cm dish (for Co‐Immunoprecipitation, Co‐IP) or 6‐cm dish (for western blotting), cultured for 36 h following transfection and then starved for 12 h in serum‐free medium. Cells were then lysed in RIPA buffer (9806S, CST, Danvers, MA, USA) to extract the total protein. Conditioned medium was concentrated using Amicon® Ultra centrifugal filters (UFC5010BK, Millipore, Billerica, MA, USA) by centrifugation at 14 000 ***g*** for 10–30 min, to a final volume of approximately 20 μL for each sample.

### 2.4 Western blotting

Approximately 30 μg of total protein was loaded, separated by 10% SDS/PAGE, and transferred to PVDF membranes (3010040001, Roche, Basel, Switzerland). The membranes were blocked with 5% skim milk powder (P0101, Maygene, Guangzhou, China) in TBST (E175‐01, GenStar, Beijing, China) for 1 h and incubated with primary antibodies, then incubated with an HRP‐conjugated secondary antibody. The specific primary and secondary antibodies used in this study are listed in Table S1. Finally, chemiluminescence was performed using SuperSignal™ West Pico PLUS chemiluminescent substrate (34579, Thermo) to visualize the protein bands of interest with a Bio‐Rad ChemiDoc Imaging System (Bio‐Rad, Hercules, CA, USA). The ratio of change in protein levels was analysed by grey‐value analysis of the target bands using imagej software (https://imagej.net/software/imagej/) compared to *beta‐actin*.

### 2.5 Co‐immunoprecipitation

Cells were seeded into a 10‐cm dish and transfected with the following pairs of plasmids: LCN2‐HA/LOXL2‐Flag, LCN2‐HA/MMP9‐Flag, LOXL2‐HA/MMP9‐Flag, LCN2‐HA/LOXL2‐HA/MMP9‐Flag, LCN2 and ΔLOXL2(1‐544aa)‐HA, LCN2 and ΔLOXL2(545‐774aa)‐HA, MMP9‐Flag and ΔLOXL2(1‐544aa)‐HA, and MMP9‐Flag and ΔLOXL2(545‐774aa)‐HA. The transfected cells were cultured for 36 h, then starved for 12 h in serum‐free medium. Then, total protein was extracted, and the conditioned medium was concentrated. The samples were subjected to co‐immunoprecipitation using anti‐DYKDDDDK magnetic agarose (A36797, Thermo) or anti‐HA magnetic beads (88836, Thermo), and placed in a rotary mixer for 30 min at room temperature. The beads were washed three times with 1× PBS, boiled, and eluted to obtain the conjugated proteins, which were subjected to anti‐Flag or anti‐HA immunoblotting analysis.

### 2.6 Mass spectrometry analysis

LCN2‐HA and LOXL2‐Flag plasmids were co‐transfected as an experimental group, and the LOXL2‐Flag plasmid was transfected as a control group in KYSE150 cells. The whole cell lysate was co‐immunoprecipitated with anti‐Flag antibody, separated by SDS/PAGE, and stained with Coomassie brilliant blue. A differentially‐expressed protein band around 25 kD was excised and subjected to in‐gel tryptic digestion, which was digested overnight at 37 °C, and the protease‐to‐protein ratio is 1 : 50 (w/w) in 10 μL of HPLC buffer A [0.1% (vol/vol) formic acid in water]. The mass spectrometry analysis process was performed as described before [14]. Resulting peptides were separated by reverse‐phase liquid chromatography on an EasynLC 1000 system (Thermo Fisher Scientific, Waltham, MA, USA) and directly sprayed into a Q Exactive mass spectrometer (Thermo Fisher Scientific). Mass spectrometry analysis was carried out in a data dependent mode with an automatic switch between a full MS and an MS/MS scan in the Orbitrap. For the full MS survey scan, the automatic gain control (AGC) target was 3e6, and the scan range was from 350 to 1750 with a resolution of 70 000. The 10 most intense peaks with charge state ≥ 2 were selected for fragmentation by higher energy collision dissociation with a normalized collision energy of 27%. MS2 spectra were acquired with 17 500 resolution. The exclusion window was set at ± 2.2 kDa. The MS/MS spectra were searched against Uniprot database (https://www.uniprot.org/) using the Mascot search engine (Matrix Science, London). Trypsin was specified as digesting enzyme. A maximum of 5 missing cleavages were allowed. An overall false discovery rate (FDR) for peptides was < 1%, and at least two unique peptides per protein. Mass tolerances for precursor ions were set at ± 0.1 Da for precursor ions and ± 0.5 Da for MS/MS.

### 2.7 Wound healing assay

A scratch wound assay was carried out to measure the distance of cell migration. After 24 h of transfection, cells were trypsinized, suspended, and adjusted to a cell density of 2.5 × 10^5^ cells·mL^−1^. Cells were inoculated into a 12‐well plate, cultured for 12 h, and then starved for 12 h in serum‐free medium. A straight‐line scratch was made on the monolayer culture at approximately 80–90% confluence using a sterile 200‐μL pipette tip. The wells were photographed under 200× magnification every 6 or 12 h with a microscope (Olympus, Tokyo, Japan). The migration ability of cells was indicated by “healed (%)” at the last time point compared to the area at 0 h. These assays were repeated, and a total of 5–8 fields were obtained.

### 2.8 Transwell migration and invasion assays

Migration and invasion assays to measure the mobility of cancer cells were performed using Transwell chambers as previously described [11]. Cells were cultured 24 h after transfection, and then starved for 12 h in serum‐free medium. The cells were digested and adjusted to 1.25 × 10^5^ cells·mL^−1^ (migration assay) or 2.5 × 10^5^ cells·mL^−1^ (invasion assay), and 400 μL of the cell suspension was added to the upper chamber. Uncoated Transwells (353097, FALCON, Los Angeles, CA, USA) were used for the migration assay, and Matrigel‐coated Transwells (356234, CORNING, Corning, NY, USA) were used for the invasion assay. Afterwards, 500 μL of 10% serum medium was added to the lower chamber. Cells were incubated for 24–36 h (migration assay) or 36–48 h (invasion assay). At the end of the culture, non‐migrated cells in the upper chamber were carefully removed with a cotton swab. The migrated cells that passed through the membrane and adhered to the lower surface of the membrane were fixed with 4% paraformaldehyde for 15 min and then stained with crystal violet for 5 min. The number of migrated or invaded cells was counted in 10 random fields photographed using a 200× microscope (IX73, OLYMPUS, Tokyo, Japan).

### 2.9 MTS assay

Transfected cells were trypsinized, suspended and adjusted to 1 × 10^5^ cells·mL^−1^ with serum‐containing medium. Then, 100 μL of the cell suspension was added to each well of a 96‐well plate (701001, NEST, Wuxi, China) in triplicate. At 0, 24, 48, 72 and 96 h after attachment, 20 μL of MTS reagent (G358B, Promega, Madison, WI, USA) was added to each well. The optical density (OD) of each well was measured using a Multiskan FC microplate reader (Thermo) after a 2‐h incubation at 37 °C. The relative growth rates were calculated by the ratio of the ODs at time points (24, 48, 72, and 96 h) relative to the OD at 0 h, suggesting their proliferation ability.

### 2.10 Flow cytometry

For flow cytometry analysis, cells were seeded into 6‐well plates and co‐transfected with the desired plasmids. After 36 h of growth, the cells were trypsinized and centrifuged (700 rpm, 10 min), and the conditioned medium was removed. The cells were resuspended in 600 μL of 1× PBS and 1.4 mL of pre‐cooled 70% ethanol, then incubated at 4 °C overnight. The next day, the cells were pelleted by centrifugation and resuspended in 500 μL of PBS, 2 μL of propidium iodide (PI) (P4864‐10ML, Sigma‐Aldrich, St. Louis, MO, USA), and 12.5 μL of RNase (2 mg·mL^−1^), followed by incubation in the dark for 30 min at room temperature. Samples were analysed on a BD Accuri C6 flow cytometer (BD Bioscience, Franklin Lakes, NJ, USA) and data analysis was performed using flowjo software (Tree Star, Ashland, OR, USA).

### 2.11 Colony‐forming assay

At 36 h after transfection, 1000 cells were seeded into 6‐well plates and cultured for about 2 weeks. When macroscopic clones were formed, cells were fixed in 4% paraformaldehyde for 20 min, stained with haematoxylin for 5 min, and photographed with a Bio‐Rad Imaging System. The number of clones was counted.

### 2.12 Zymography analysis

MMP9 activity was measured with gelatin zymography as previously described [11]. Conditioned medium from transfected cells was concentrated and subjected to SDS/PAGE electrophoresis with 1% gelatin. Following electrophoresis, the separation gel was washed twice in washing buffer (2.5% Triton X‐100) for 30 min each wash, then incubated in incubation buffer [40 mm Tris–HCl (pH 8.0), 10 mm CaCl_2_] at 37 °C for 24 h. The gel was stained with 0.1% Coomassie blue dye for 1 h, destained, and fixed. The proteolytic activities of MMP9 and its complex were detected as a clear white zone against a Coomassie blue‐stained gel background.

### 2.13 Fluorescent matrix degradation assay

Glass coverslips were coated with 0.01% poly‐L‐lysine (P8920‐100ML, Sigma‐Aldrich), air‐dried overnight, and fixed with 0.5% glutaraldehyde. Fibronectin HiLyte 488 (FNR02‐A, Cytoskeleton, Denver, CO, USA) was added dropwise to each glass coverslip. The coverslips were then transferred to a 24‐well plate and incubated at 37 °C for 1 h. Cells were suspended following trypsinization and added to the glass coverslips. After 48 h, cells on each glass coverslip were fixed, permeabilized and labelled with Acti‐Stain™ 555 fluorescent phalloidin (PHDH1, Cytoskeleton), followed by mounting in anti‐fluorescence attenuating reagent (S2110, Solarbio, Beijing, China). For the determination of matrix degradation, the coverslips were photographed using a laser confocal microscope (ZEISS LSM 800, Carl Zeiss, Jena, Germany).

### 2.14 3D cell *in vitro* culture

Matrigel™ (BD Biosciences, Bedford, MA, USA) was thawed on ice, and diluted 1 : 1 with serum‐free cold cell culture media. The transfected cells were trypsinized and adjusted to 1 × 10^6^ cells·mL^−1^, and then 50 μL of diluted Matrigel and 10 μL of the cell suspension were mixed in a 6‐well plate, and incubated at 37 °C for 30 min. After solidifying, 3 mL of 10% fresh serum medium was added, and cells were photographed under a 200× inverted microscope every 3–6 h. The length of filopodia around the cell was quantified using ImageJ software.

### 2.15 Immunofluorescence

Cells were seeded into 6‐well plates and co‐transfected with the desired plasmids. After 36 h, the cells were trypsinized, inoculated into a 24‐well plate containing coverslips, and cultured for an additional 12–24 h. The cells were fixed with 4% paraformaldehyde, permeabilized with 0.1% Triton X‐100 (T0694‐100ML, AMRESCO, Solon, OH, USA) for 3 min at room temperature, then blocked with 5% donkey serum at room temperature for 1 h. Primary antibodies were added and incubated at 4 °C overnight, including antibodies against LCN2, LOXL2, MMP9, HA, Flag, and profilin 1. Fluorescent secondary antibody was dropped onto the coverslips and incubated at room temperature for 1 h in the dark. The fluorescent secondary antibodies used herein included Acti‐Stain™ 555 fluorescent phalloidin (Cat. # PHDH1, Cytoskeleton), Acti‐Stain™ 488 fluorescent phalloidin (Cat. # PHDG1, Cytoskeleton), Alexa Fluor 488 DAM ReadyProbes reagent with NZ‐derived BSA (R37114, Invitrogen, Carlsbad, CA, USA), and Alexa Fluor 594 DAR ReadyProbes reagent with NZ‐derived BSA (R37119, Invitrogen). The slides were photographed with a laser confocal microscope (ZEISS LSM800). The fluorescence intensity of profilin 1 was quantified using ImageJ software.

### 2.16 Xenograft assay in nude mice

Animal experiments were conducted in accordance with protocols approved by the Animal Ethics Committee of Shantou University Medical College (No. SUMC2016‐037). All mice were treated humanely, housing in a specific pathogen‐free environment and allowing for adaption to their environment before experiments. Four‐week‐old nu/nu male nude mice (Beijing Vital River Laboratory Animal Technology Co., Ltd, Beijing, China) were randomized into 10 groups (five mice/group), and their footpads or subcutaneous layer were inoculated with 1 × 10^6^ cells (100 μL × 10^7^ cell·mL^−1^) of each stably transfected KYSE150 cell line or wildtype KYSE150 cells. The general behaviour of the mice was monitored, and tumour size was measured every 3 days for a total of 30 days. For drug treatment experiments, nude mice injected subcutaneously were observed for the first 3 days, and then intraperitoneal injections of 200 mg·kg^−1^ deferoxamine mesylate (DFOM) (HY‐B0988, MCE, Shanghai, China) were administered daily, for a total of 14 days. At the end of the experiment, the nude mice were euthanized and xenograft tumours, including footpad primary tumours, popliteal‐infiltrated tumours, popliteal lymph nodes were dissected, weighed, and analysed as described previously [11]. The tumour sizes and lymph node volumes were calculated by the formula: (width^2^ × length)/2.

### 2.17 Statistical analysis

spss 13.0 (SPSS, Inc., Armonk, NY, USA) was used for data analyses, and two‐group comparisons were performed using Student's *t* tests. One‐way analysis of variance was used to assess the differences between groups when more than two groups were compared. Differences were considered statistically significant at * for *P* < 0.05, ** for *P* < 0.01 and *** for *P* < 0.001. For the correlation test of the two groups of data, Pearson correlation analysis was used. Experimental data are presented as the means ± SD of at least three independent experiments.

## 3 Results

### 3.1 Protein–protein interaction network and cell model construction

To identify the proteins that interact with LOXL2, we performed co‐IP using LOXL2 as bait followed by LC–MS analysis. Mass spectrometry results showed that multiple LCN2 protein peptides were identified from a differentially‐expressed 25 kDa band from *LOXL2*‐overexpressing cells. The PEP values of the experimental group peptides were far < 0.1, and the cumulative coverage was as high as 42.3%, indicating the results were highly credible (Table S2). The secondary ion mass spectrum of the VPLQQNFQDNQFQGK proteolysis product of LCN2 protein is shown in Fig. S1A. The peaks represent the secondary ion peaks of the peptide measured from the mass spectrum. These results indicated that LOXL2 and LCN2 interacted to form a complex.

Next, to gain a complete view of the interactome between LCN2 and LOXL2, all literature reporting interacting proteins was obtained from HPRD (http://www.hprd.org/) and BioGRID (http://thebiogrid.org/), and were processed and merged into a “parent network” as previously described [18]. The parent network was entered into the Cytoscape program to construct a secondary protein interaction sub‐network using LCN2 and LOXL2 as the seed proteins. LCN2 was linked with LOXL2 by at least five pairs of partner proteins, further supporting that LCN2 interacted directly or physically with LOXL2 (Fig. S1B). To understand their correlation in ESCC clinical samples, the Pearson correlations for LCN2/MMP9, LCN2/LOXL2, and MMP9/LOXL2 in the public GSE53625 dataset were examined. A significant correlation was found for LCN2/MMP9 and MMP9/LOXL2 (Fig. S1C).

The endogenous expression level of *LCN2* and *LOXL2* was examined in eight oesophageal cancer cell lines, with human 293T as a control. KYSE410 and KYSE150 cells showed relatively low endogenous LCN2 and LOXL2 levels and were selected as the cell models for subsequent experiments (Fig. S1D). KYSE410 and KYSE150 cells were co‐transfected with LCN2‐HA/LOXL2‐Flag, LCN2‐HA/MMP9‐Flag, or LOXL2‐HA/MMP9‐Flag plasmid pairs, or the corresponding vector plasmids. High expression of the target proteins was observed in KYSE410 and KYSE150 cells after transfection (Fig. S1E).

Three tissue microarrays were performed to detect the expression of *LCN2*, *MMP9* and *LOXL2* in 20 pairs of ESCC clinical samples using immunohistochemistry (IHC) method. The results show that *LCN2*, *MMP9* and *LOXL2* were highly co‐expressed at protein level in 80% (16/20) ESCC clinical samples, and they illustrated a strong cytoplasm staining (Fig. S2).

### 3.2 The interaction patterns of intracellular and extracellular LCN2/LOXL2/MMP9

**Fig. 1:**
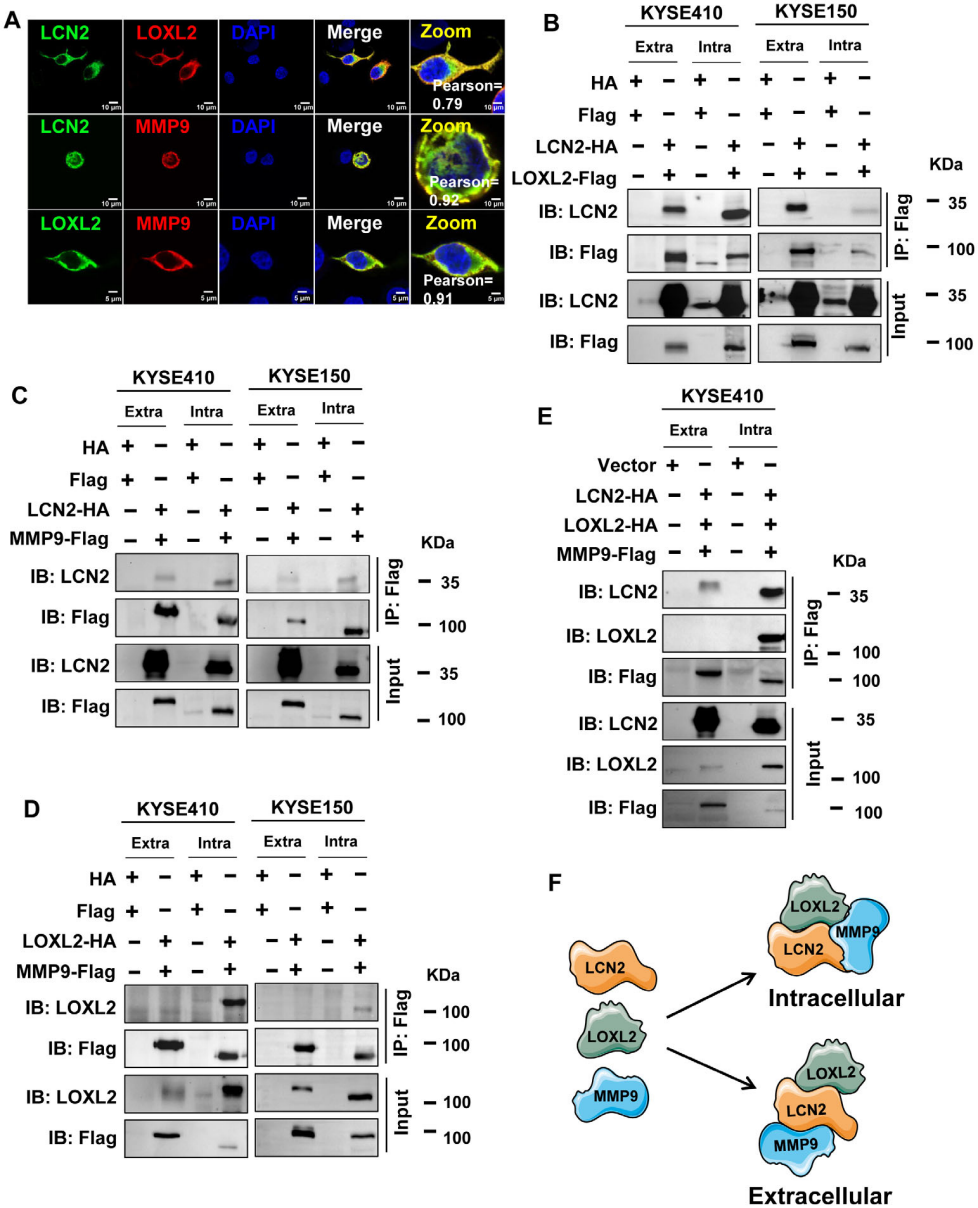
Identification of LCN2/LOXL2/MMP9 protein–protein interaction patterns. (A) Immunofluorescence was applied to observe the endogenous co‐localization of LCN2 with LOXL2, LCN2 with MMP9, and LOXL2 with MMP9 in KYSE150 cells. Immunofluorescence experiment was performed once and representative images with scale bars, 10 μm (top two panels) or 5 μm (bottom panel). For the interaction experiments, several pairs of plasmids were co‐transfected into KYSE410 and KYSE150 cells: LCN2‐HA/LOXL2‐Flag (B), LCN2‐HA/MMP9‐Flag (C), and LOXL2‐HA/MMP9‐Flag (D). Co‐IP was used to determine the interactions between LCN2‐HA, LOXL2‐HA, and MMP9‐Flag in KYSE410 cells (E). After 48 h of transfection, total protein was extracted and the cell‐conditioned medium was concentrated, followed by co‐IP with the indicated antibodies. Representative images were shown with at least three times in (B–E). (F) Schematic diagram of the intracellular and extracellular protein–protein interactions between LCN2, LOXL2, and MMP9.

The protein–protein interaction network (PPIN) results showed that MMP9 interacted with both LCN2 and LOXL2. To confirm this, the intracellular interactions between the three proteins were detected by immunofluorescence. The results revealed that in the KYSE150 cell line, LCN2 and LOXL2, LCN2 and MMP9, and LOXL2 and MMP9 were all co‐localized after they were overexpressed, with Pearson’ correlation coefficients of 0.79, 0.92, and 0.91, respectively (Fig. 1A). This suggested that the ternary complex of LCN2/LOXL2/MMP9 existed in oesophageal cancer cells. To further confirm the presence of LCN2/LOXL2/MMP9 ternary complexes in ESCC cells, co‐immunoprecipitation using whole cell lysate was performed after co‐transfection of LCN2‐HA/LOXL2‐Flag, LCN2‐HA/MMP9‐Flag, or LOXL2‐HA/MMP9‐Flag.

Considering that LCN2, LOXL2, and MMP9 are all secreted proteins, the cell culture supernatant after transfection was also collected and concentrated to detect the extracellular interactions of these three proteins. The results showed that in both KYSE410 and KYSE150 cells, protein–protein interaction between LCN2 and LOXL2, and between LCN2 and MMP9, occurred both intracellularly and extracellularly (Fig. 1B,C). However, the protein–protein interactions between LOXL2 and MMP9 occurred only intracellularly (Fig. 1D). Next, we co‐overexpressed LCN2‐HA/LOXL2‐HA/MMP9‐Flag in KYSE410 cells and carried out co‐immunoprecipitation using the whole cell lysates and culture supernatants. We found protein–protein interactions between MMP9 and LCN2 both intracellularly and extracellularly, whereas the protein–protein interaction between MMP9 and LOXL2 was identified only intracellularly (Fig. 1E). Moreover, endogenous interactions of LCN2 and LOXL2 and of LCN2 and MMP9 were confirmed in KYSE150 and KYSE180 ESCC cell lines (Fig. S3). A schematic of the intracellular and extracellular protein–protein interaction pattern of LCN2, LOXL2, and MMP9 is shown in Fig. 1F.

### 3.3 LCN2/LOXL2/MMP9 protein–protein interaction promotes migration and invasion of ESCC cells

**Fig. 2:**
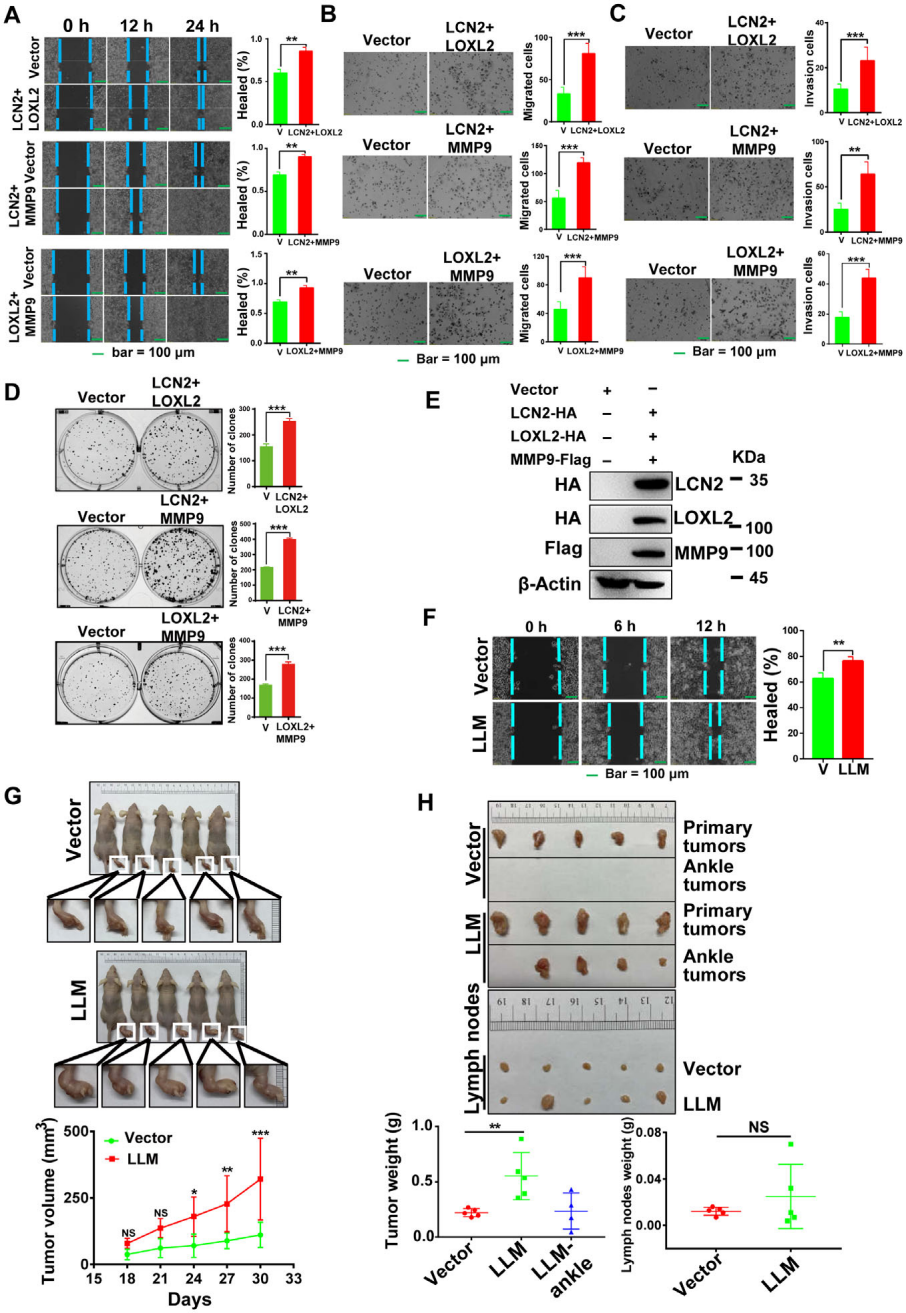
Protein–protein interaction between LCN2/LOXL2/MMP9 promotes migration and invasion of ESCC cells. *LCN2*/*LOXL2*, *LCN2*/*MMP9*, or *LOXL2*/*MMP9* were co‐transfected into KYSE150 cells. Wound‐healing (A) and migration assays (B) were used to detect the migration of oesophageal cancer cells. (C) A Transwell assay with Matrigel‐coated membranes was used to study the invasive capacity of ESCC cells. Bar = 100 μm in (A–C). (D) Colony‐forming assays were used to detect the proliferation of ESCC cells. (E) Western blotting verified the stable expression of LCN2‐HA/LOXL2‐HA/MMP9‐Flag in KYSE150 cells. Representative images were shown with at least three times. (F) Migration of oesophageal cancer cells was detected by wound‐healing assays in *LCN2*/*LOXL2*/*MMP9*‐overexpressing KYSE150 cells. Bar = 100 μm in (F). Representative images were shown with at least three times in (A–F). (G) Nude mice were injected in their footpads with 100 μL of KYSE150 (1 × 10^7^ cells·mL^−1^ in serum‐free medium) stably expressing *LCN2*/*LOXL2*/*MMP9* or the empty vector, and the tumour volume was measured every 3 days for a total of 30 days. The lower panel graph shows representative images and tumour volumes measured in xenograft nude mice at the specified time points (*N* = 5, error bars represent SD). (H) Top, images of the primary tumours of the footpad, metastatic tumours of the ankle, and distal lymph nodes that were excised; bottom, average tumour weights measured after the end of the experiment (*N* = 5, error bars represent SD). The scale units in the image of (G, H) is cm. LLM means LCN2/LOXL2/MMP9. Statistical differences were analysed using Student's *t*‐tests for two groups. Error bars represent SD from triplicate experiments in (A–D, F). *P < 0.05; **P < 0.01; ***P < 0.001; NS, not significant.

LCN2, LOXL2, and MMP9 formed a ternary complex in oesophageal cancer cells, but the biological function of the ternary complex remained to be explored. In the KYSE150 cell line, wound healing experiments showed that co‐overexpression of *LCN2*/*LOXL2*, *LCN2*/*MMP9* and *LOXL2*/*MMP9* increased the migration of ESCC cells (Fig. 2A). Moreover, both Transwell assays and invasion assays showed that overexpression of *LCN2*/*LOXL2*, *LCN2*/*MMP9* and *LOXL2*/*MMP9* enhanced the migration and invasion of oesophageal cancer cells (Fig. 2B,C). Similar results were also obtained with the KYSE410 cell line (Fig. S4A–C). Compared to the group solely transfected with LCN2, either co‐transfection of *LCN2*/*LOXL2* or co‐transfection of all three proteins significantly increased the migration and invasion of oesophageal cancer cells (Fig. S4D).

In addition to migration and invasion, we also investigated the functional role of the LCN2/LOXL2/MMP9 ternary complex in cell proliferation. Colony‐forming assays showed that overexpression of *LCN2*/*LOXL2*, *LCN2*/*MMP9* and *LOXL2*/*MMP9* enhanced cell colony formation in the KYSE150 cell line (Fig. 2D). Similar results were obtained with the KYSE410 cell line (Fig. S4E). MTS assay results showed that proliferation of ESCC cells in the experimental group did not change significantly after their overexpression in either KYSE410 or KYSE150 cells (Fig. S4F,G). In the KYSE150 cell line, flow cytometry showed no significant cell cycle change in the experimental group (Fig. S4H).

To obtain a stably overexpressing cell line, the Neon™ transfection system (Thermo) was used to electroporate the LCN2‐HA/LOXL2‐HA/MMP9‐Flag (abbreviated as LLM) plasmids into the KYSE150 cell line, followed by 2 weeks of G418 and hygromycin selection. Western blotting showed that a cell line stably overexpressing all three proteins was successfully established (Fig. 2E). Consistently, wound healing experiments showed that LLM increased the migration of oesophageal cancer cells (Fig. 2F). To study the effect of LLM on ESCC tumour progression *in vivo*, we separately injected the cells stably overexpressing the ternary LLM complex and control cells into the footpads of nude mice. Compared with the control group, LLM cells displayed enhanced tumour progression (Fig. 2G). The anatomical results showed that four out of five nude mice overexpressing LLM developed tumour metastasis, in which the tumour cells migrated from the footpad to the ankle, and the lymph nodes of two nude mice were swollen (Fig. 2H).

### 3.4 LCN2/LOXL2 and MMP9/LOXL2 interactions depend on the SRCR domains

**Fig. 3:**
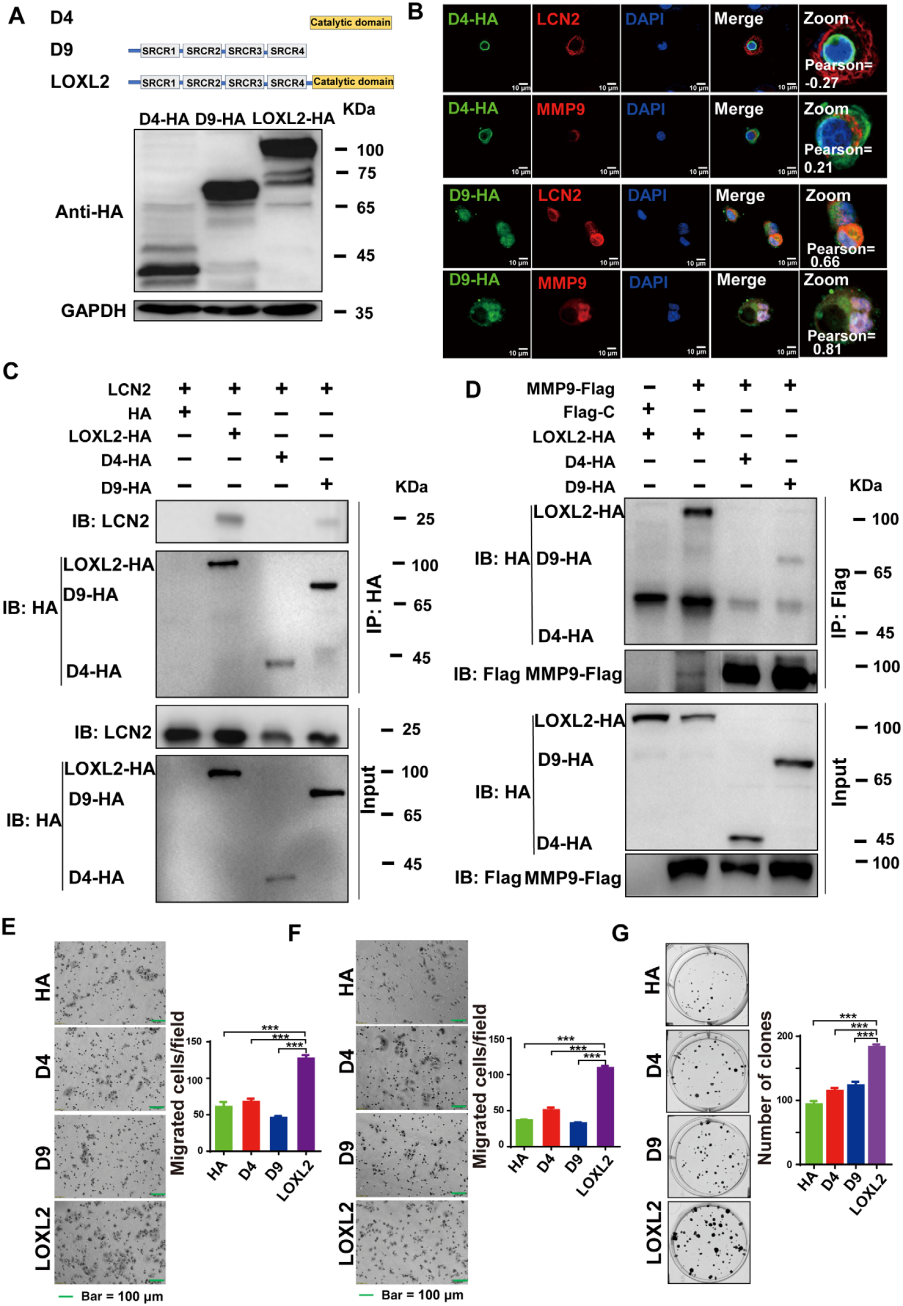
Identification of LOXL2/LCN2 and LOXL2/MMP9 protein–protein interaction domains. (A) Schematic diagram of truncated *LOXL2* plasmid construction and their expression as identified by western blot. (B) In KYSE150 cells, D4‐HA and D9‐HA were co‐transfected with *LCN2* or *MMP9*. Their co‐localizations were observed by immunofluorescence. Scale bars, 10 μm. (C) LCN2/HA‐V, LCN2/LOXL2‐HA, LCN2/D4‐HA, or LCN2/D9‐HA were co‐transfected. Co‐IP was used to identify the domain necessary for LCN2 and LOXL2 protein–protein interaction. (D) LOXL2‐HA/Flag‐C, LOXL2‐HA/MMP9‐Flag, MMP9‐Flag/D4‐HA, or MMP9‐Flag/D9‐HA were co‐transfected to identify the interaction domain between MMP9 and LOXL2. Migration (E) and invasion (F) assays were used to compare the effects of truncated *LOXL2* on the migration and invasion of cancer cells. Bar = 100 μm in (E, F). (G) A colony‐forming assay was used to compare the effects on the proliferation of carcinoma cells. Each experiment was repeated at least three times. Statistical differences were analysed using Student's *t*‐tests for two groups. Error bars represent SD from triplicate experiments in (E–G). Representative images were shown with at least three times in (A–G). ***P < 0.001.

To identify the specific domains for LOXL2/LCN2 and LOXL2/MMP9 protein–protein interactions in the ternary complex, two truncated LOXL2 plasmids were constructed, ΔLOXL2‐HA [545–774 aa; lacking the N‐terminal scavenger receptor cysteine‐rich (SRCR) domains, denoted D4‐HA], and ΔLOXL2‐HA (1–544 aa; lacking the C‐terminal amine oxidase domain, denoted D9‐HA). First, we confirmed that the two plasmids were able to be expressed in KYSE150 cells (Fig. 3A). For endogenous interactions, immunofluorescence showed that in the KYSE150 cell line, D9‐HA was co‐localized with LCN2 or MMP9, with Pearson correlation coefficients of 0.66 and 0.81, respectively (Fig. 3B). However, D4‐HA did not appear to co‐localize with LCN2 or MMP9 in this cell line. Confocal Z‐stack analysis was applied to scan different sections of transfected cells to confirm their co‐localization (Fig. S5). Furthermore, co‐immunoprecipitation results showed that the protein–protein interactions between LOXL2 and either LCN2 or MMP9 disappeared when the SRCR domains of LOXL2 were deleted (Fig. 3C,D). These results suggested that the protein–protein interactions for LCN2/LOXL2 and MMP9/LOXL2 both depended on the SRCR domains of LOXL2. Additionally, the biological roles of the N‐terminus and C‐terminus of LOXL2 were investigated. Transwell and invasion assays showed that the two truncated plasmids (D4 and D9) reduced the ability of ESCC cells to migrate and invade compared to full‐length LOXL2 (Fig. 3E,F). Similar results were also observed in colony formation assays (Fig. 3G).

### 3.5 The LCN2 inhibitor DFOM inhibits the migration and tumour growth of ESCC cells

**Fig. 4:**
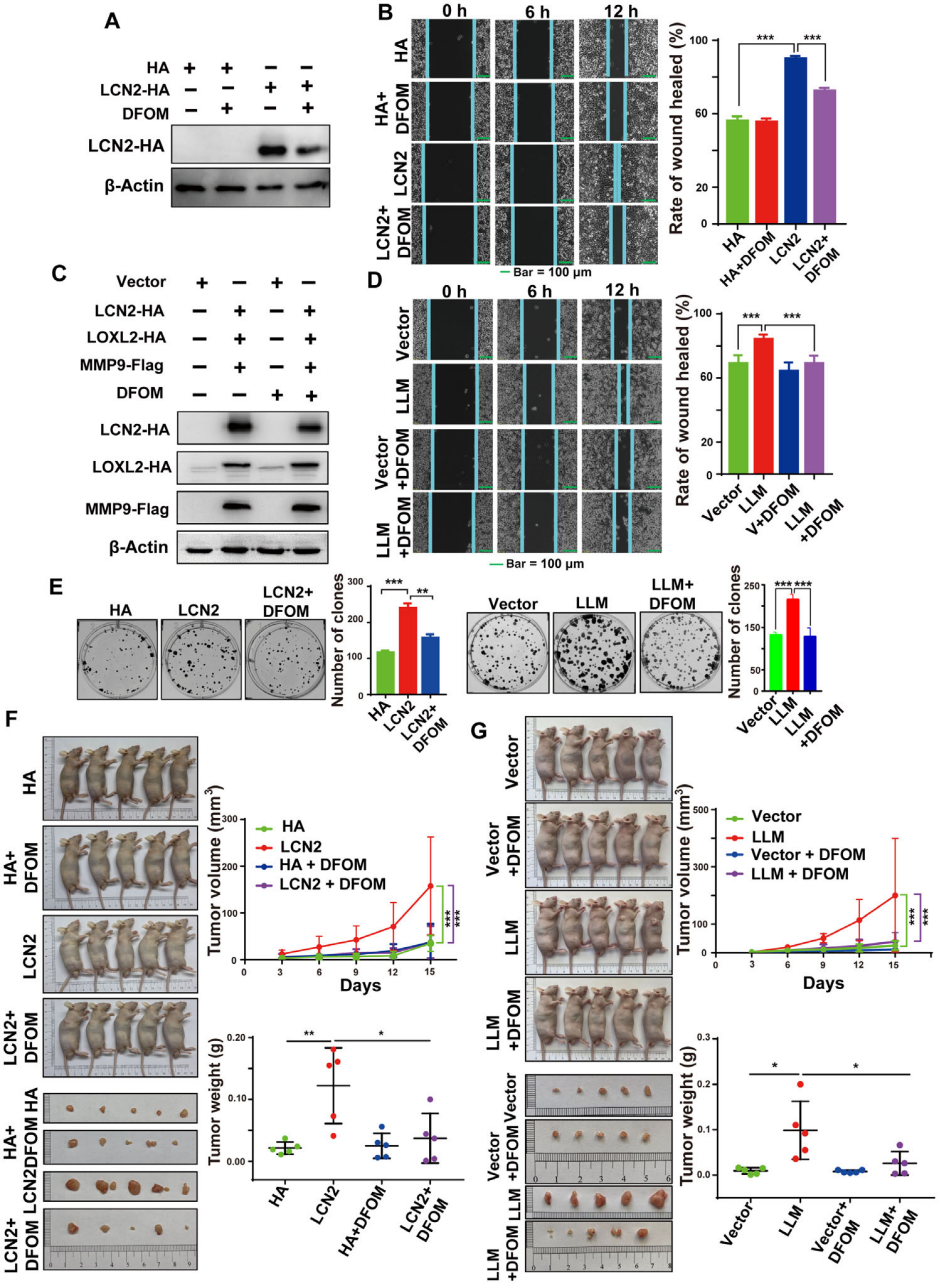
DFOM inhibits the migration and tumour growth of oesophageal cancer cells. In KYSE150 cells, *LCN2* or *LCN2*/*LOXL2*/*MMP9* (LLM) was overexpressed and then cells were treated with DFOM (30 μm) for 24 h. Western blotting was used to detect the expression level of *LCN2* (A, C), and a wound‐healing assay was used to detect cell migration (B, D). Bar = 100 μm in (B, D). Representative images were shown with at least three times in (A, C). (E) Colony‐formation assays were used to detect cell proliferation. *LCN2* or *LCN2*/*LOXL2*/*MMP9* (LLM) was overexpressed and treated with DFOM (30 μm) for 7 days with a total of 14 days of culture. Representative images were shown with at least three times in (A–E). Additionally, 100 μL of KYSE150 (1 × 10^7^ cells·mL^−1^ in serum‐free medium) stably overexpressing *LCN2* (F) or *LCN2*/*LOXL2*/*MMP9* (LLM) (G) was injected into the right armpit of nude mice. The drug treatment began 3 days after injection. DFOM (200 mg·kg^−1^) or saline (0.9% NaCl) was injected into the abdominal cavity for a total of 14 days, the nude mice were euthanized, and tumours were excised. Representative nude mice and anatomical tumours are shown, and the nude mouse tumour volume at the specified time points and the final tumour weights were measured (*N* = 5, error bars represent SD). The scale units in the image of (F, G) is cm. LLM means *LCN2*/*LOXL2*/*MMP9*. Statistical differences were analysed using Student's *t*‐tests for two groups, one‐way analysis of variance for more than two groups. Error bars represent SD from triplicate experiments in (B, D, E). *P < 0.05; **P < 0.01; ***P < 0.001.

Deferoxamine mesylate (DFOM) is an iron chelator that binds free ferric iron and is mainly used to treat diseases caused by iron overload [19]. KYSE150 cells were treated with 0, 5, 10, 25, 50, 100, and 200 μm DFOM. After 48 h, an MTS assay was performed, and the IC50 was calculated to be 31 μm (Fig. S6). Therefore, 30 μm (for the convenience of its dilution) DFOM was used for subsequent experiments. DFOM decreased the expression level of *LCN2* in KYSE150 cells (Fig. 4A). Wound healing experiments showed that *LCN2* overexpression‐mediated enhancement of ESCC cell migration was reduced by DFOM treatment (Fig. 4B). Compared with the stable overexpression of LLM, these results showed that the expression of *LCN2* was significantly reduced after DFOM treatment, indicating that DFOM may disrupt the formation of the ternary complex LLM or reduce its expression (Fig. 4C). Compared with the empty vector group, the migration of ESCC cells was significantly promoted by LLM, which decreased after DFOM treatment (Fig. 4D). Colony‐forming assays showed that LCN2 or LLM promoted the proliferation of single ESCC cells, and this effect was inhibited by DFOM treatment (Fig. 4E). To study the effect of LCN2 and the LLM ternary complex on tumour progression of ESCC *in vivo*, as well as the anti‐tumour effect of DFOM *in vivo*, a xenograft model was used. Compared with the control group, LCN2 promoted ESCC tumour growth *in vivo*. However, after intraperitoneal administration of DFOM, it was found that both the tumour volume and weight were significantly reduced (Fig. 4F). Similarly, the LLM ternary complex promoted tumour progression *in vivo* compared to the vector group. Consistently, tumour growth was inhibited, and the tumour volume and weight were also reduced after DFOM treatment (Fig. 4G).

### 3.6 LCN2/LOXL2/MMP9 promoted the degradation of the ECM

**Fig. 5:**
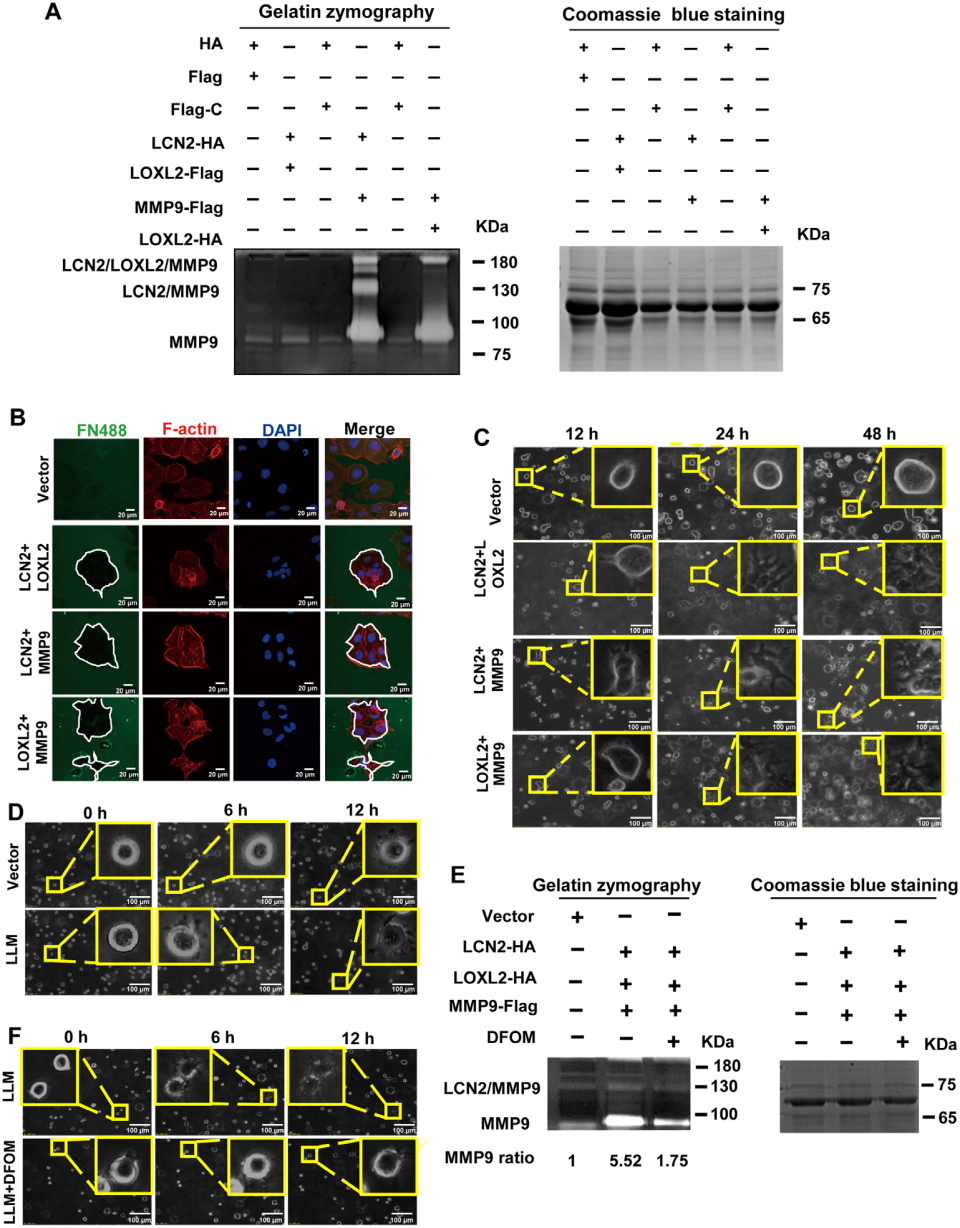
LCN2, LOXL2, and MMP9 protein–protein interactions promote extracellular matrix degradation. (A) Zymography (left panel) was applied to detect enzyme activities of MMP2 and MMP9, after the co‐overexpression of *LCN2*/*LOXL2*, *LCN2*/*MMP9*, or *LOXL2*/*MMP9*, as indicated. The amount of loaded total protein is shown in the right panel. (B) Fluorescent matrix degradation was performed to observe ECM degradation. The white curvilinear frame indicates the matrix degradation area. FN488 fluorescent matrix gel is shown in green, F‐Actin is stained red, and nuclei are stained blue with DAPI. Scale bars, 20 μm. (C, D) *LCN2*/*LOXL2*, *LCN2*/*MMP9*, *LOXL2*/*MMP9*, and *LCN2*/*LOXL2*/*MMP9* (LLM) were transfected as indicated. Overexpressing cells were mixed with Matrigel to construct a 3D culture model. Degradation of the matrix and formation of cell filopodia were observed at different time points. (E) *LCN2*/*LOXL2*/*MMP9* was overexpressed, and then cells were treated with DFOM (30 μm) for 24 h. The supernatant was collected and the enzymatic activity of MMP9 was detected by zymography. (F) Cells overexpressing *LCN2*/*LOXL2*/*MMP9* (LLM) were mixed with Matrigel to construct a 3D culture model, to observe the effect of DFOM on the degradation of the matrix and the formation of filopodia. Scale bars, 100 μm in (C, D, F). Representative images were shown with at least three times in (A–E).

The LCN2/LOXL2/MMP9 ternary complex promoted the migration and invasion of ESCC cells, but the specific molecular mechanism was still unclear. In the experimental groups that overexpressed *LCN2*/*MMP9* or *LOXL2*/*MMP9*, the ability of MMP9 to degrade gelatin increased in zymography experiments (Fig. 5A). It was also observed that gelatin degradation occurred at 130 kDa in extracts of LCN2/MMP9 co‐overexpressing cells, which corresponded to the LCN2/MMP9 complex. In cells co‐overexpressing *LCN2*/*MMP9* or *LOXL2*/*MMP9*, gelatin degradation was also seen at around 200 kDa, which coincided with the predicted molecular weight of the LCN2/LOXL2/MMP9 ternary complex. We presumed that when co‐overexpressing *LCN2*/*MMP9* or *LOXL2*/*MMP9*, a LCN2/LOXL2/MMP9 ternary complex synergistically degraded the matrix, which promoted the migration and invasion of ESCC cells (Fig. 5A).

Next, visualization of ECM degradation in living cells was performed using fluorescent matrix degradation assays. In KYSE150 cells co‐overexpressing *LCN2*/*LOXL2*, *LCN2*/*MMP9*, or *LOXL2*/*MMP9*, the ability to degrade fluorescent Matrigel was enhanced, as a significant black hole was formed after degradation, whereas Matrigel degradation was barely observed in the control cells (Fig. 5B). Three sets of co‐overexpressing cells were scanned from top to bottom using the Z‐stack function of the confocal microscope. The results confirmed that cells co‐overexpressing *LCN2*/*LOXL2*, *LCN2*/*MMP9*, or *LOXL2*/*MMP9* degraded more fluorescent Matrigel around the cells (Fig. S7).

The growth environment of tumour cells *in vivo* is three‐dimensional. For this reason, we further characterized the ability of tumour cells to degrade the ECM in a 3D cell culture model. Cells in the control group displayed a round and smooth morphological structure, except for the enlargement of the cell body itself. In the *LCN2*/*LOXL2*‐, *LCN2*/*MMP9*‐, or *LOXL2*/*MMP9*‐overexpressing groups, in addition to enlargement of the cell body, the ability of cells to degrade Matrigel was enhanced as more Matrigel surrounding the cells was digested. As the culture time was prolonged, elongation and branching of filopodia around the cells became clearly visible (Fig. 5C). In the stably overexpressing *LCN2*/*LOXL2*/*MMP9* cell line, a similar result was obtained in which LLM significantly degraded the Matrigel and promoted the extension of filopodia (Fig. 5D).

MMP9 activity in the ternary complex was examined. The zymography results indicated that the ternary complex LLM significantly enhanced the enzymatic activity of MMP9 to degrade gelatin and form a distinct degradation band, while DFOM treatment significantly weakened the enzymatic activity of MMP9 (Fig. 5E). The results of 3D cell culture showed that after DFOM treatment of ESCC cells, the ability of the LLM complex to degrade Matrigel was reduced along with the length of filopodia (Fig. 5F). The quantification of the length of filopodia in Fig. 5C,D,F are indicated in Fig. S8. Clear differences were observed between the control group and the overexpression group, which was inhibited by the iron chelator DFOM.

Because the interaction of LCN2/LOXL2 and MMP9/LOXL2 was shown to depend on the SRCR domains of *LOXL2*, we further evaluated the importance of the SRCR domains. The formation of filopodia was observed in cells transfected with truncated *LOXL2* and the full‐length *LOXL2* after 48 h (Fig. S9). The number and extension length of filamentous pseudopods of cells transfected with full‐length *LOXL2* were greater than those in cells transfected with the catalytic domain (D4) (Fig. S9A,B). Minimal change was observed in cells transfected with the SRCR domain (D9), which indicated that the SRCR domains of LOXL2 play an important role in ECM degradation and filopodia formation.

### 3.7 LCN2, LOXL2, and MMP9 induce cytoskeletal microfilament remodelling

**Fig. 6:**
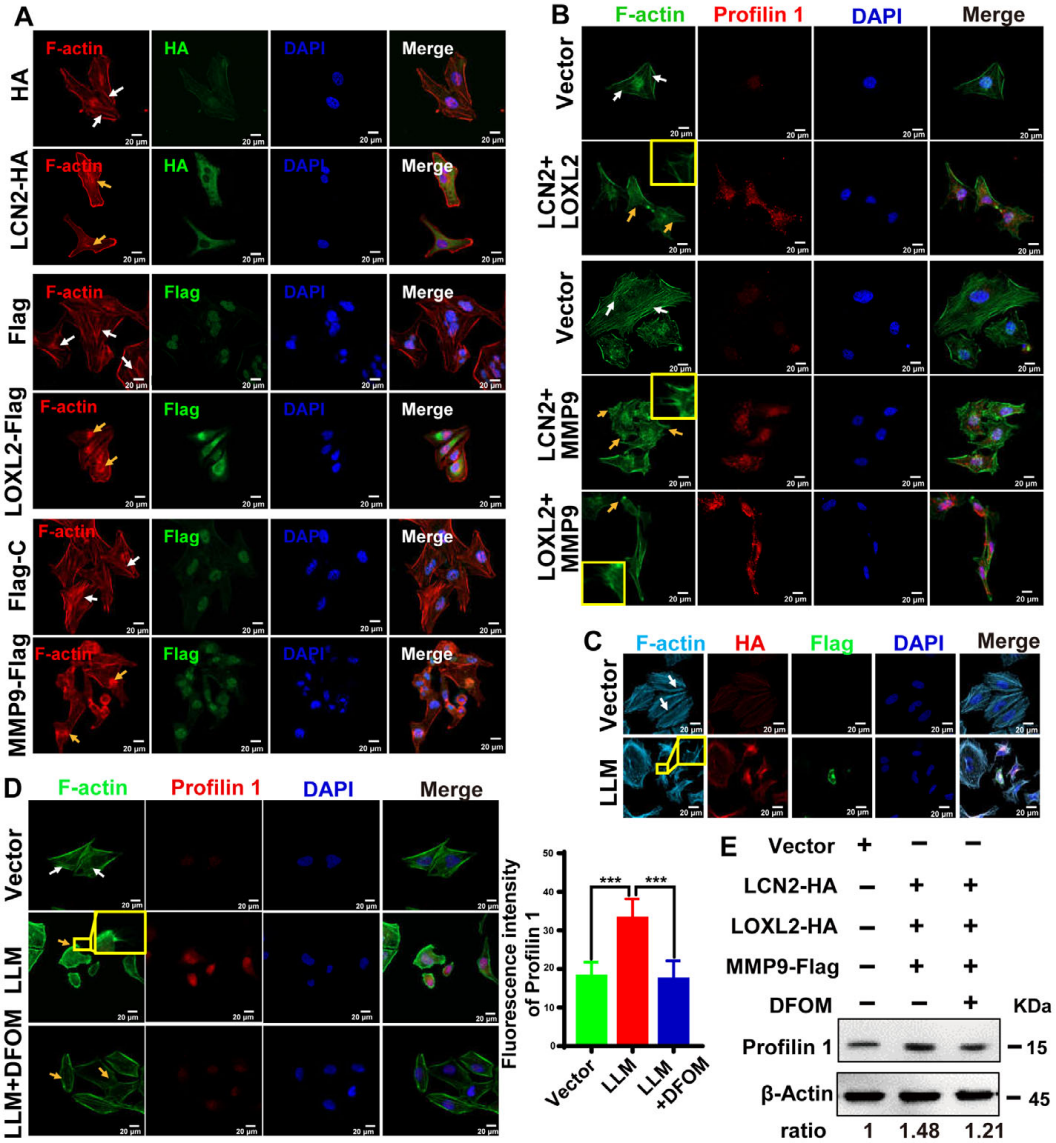
LCN2/LOXL2/MMP9 protein–protein interaction promotes microfilament remodelling. F‐Actin and profilin 1 were labelled via immunofluorescence to detect their expression and distribution in cancer cells after transfection of *LCN2*, *LOXL2*, or *MMP9*, individually (A) and in combination (B), to analyse the re‐arrangement of the Actin cytoskeleton. (C) LCN2‐HA/LOXL2‐HA/MMP9‐Flag was overexpressed in KYSE410 cells. HA and Flag were fluorescently labelled using immunocytochemistry to observe the expression of the ternary complex, and F‐Actin was fluorescently labelled to observe the changes of the cytoskeleton. (D) Cells overexpressing *LCN2*/*LOXL2*/*MMP9* (LLM) were treated with DFOM for 24 h, and changes in F‐Actin and the expression and distribution of profilin 1 were observed (left panel). The white arrow points to the orderly microfilament skeleton in the cell, the orange arrow indicates the disordered cytoskeleton or the elongated filopodia, and the yellow frame represents the partially enlarged view. The fluorescence intensity of profilin 1 was quantified using ImageJ software (right panel). Statistical differences were analysed using Student's *t*‐tests for two groups. Error bars represent SD from triplicate experiments in (D). ***P < 0.001. Scale bar, 20 μm. (E) Western blot was used to detect the effect of DFOM on the expression of profilin 1 after LCN2‐HA/LOXL2‐HA/MMP9‐Flag overexpression. The relative fold changes were quantified by ImageJ software. Representative images were shown with at least three times in (A–E).

Tumour cell migration and invasion usually involve rearrangement of the cellular microfilament system. To better observe changes in the actin cytoskeleton of ESCC cells, F‐actin in KYSE410 cells was fluorescently labelled after overexpression of the target proteins. The results showed that stress fibres in the control cells were arranged in regular bundles, while the stress fibres in the overexpression group were more disordered, suggesting that overexpression of *LCN2*, *LOXL2* and *MMP9* promoted cell migration by remodelling the cell microfilament cytoskeleton (Fig. 6A). Profilin 1 (PFN1) is a member of the profilin family of small actin‐binding proteins that play an important role in actin dynamics by regulating actin polymerization in response to extracellular signals [20]. To observe the actin arrangement, F‐actin and profilin 1 were simultaneously fluorescently labelled after *LCN2*/*LOXL2*, *LCN2*/*MMP9*, or *LOXL2*/*MMP9* transfection. The microfilament skeleton in the three overexpressing groups was more disordered compared with the control group (Fig. 6B). The protein level of profilin 1 increased and accumulated in the cytoplasm and at the leading edge of the cell. These results indicated that LCN2, LOXL2, and MMP9 promoted cell migration by enhancing *profilin 1* expression and promoting microfilament rearrangement (Fig. 6B).

In the KYSE410 cell line, LCN2‐HA/LOXL2‐HA/MMP9‐Flag was overexpressed, and the HA and Flag tags were fluorescently labelled. It was observed that *LCN2*, *LOXL2*, and *MMP9* were all successfully over‐expressed. Compared with the empty vector group, the cytoskeleton of cells overexpressing LLM was disordered, and the number of filopodia significantly increased (Fig. 6C). Next, we aimed to analyse whether DFOM treatment could rescue the cytoskeleton disorder. We found the fluorescence intensity of profilin 1 increased in LLM‐overexpressing cells, but decreased after DFOM treatment and was accompanied by restoration of the cytoskeleton organization to an orderly state (Fig. 6D). These results suggested that the LLM ternary complex enhanced the expression of *profilin 1*. To confirm this, western blotting showed that the expression of *profilin 1* decreased after DFOM treatment (Fig. 6E).

### 3.8 LCN2/LOXL2/MMP9 activate the FAK/AKT/GSK3β signalling pathway

**Fig. 7:**
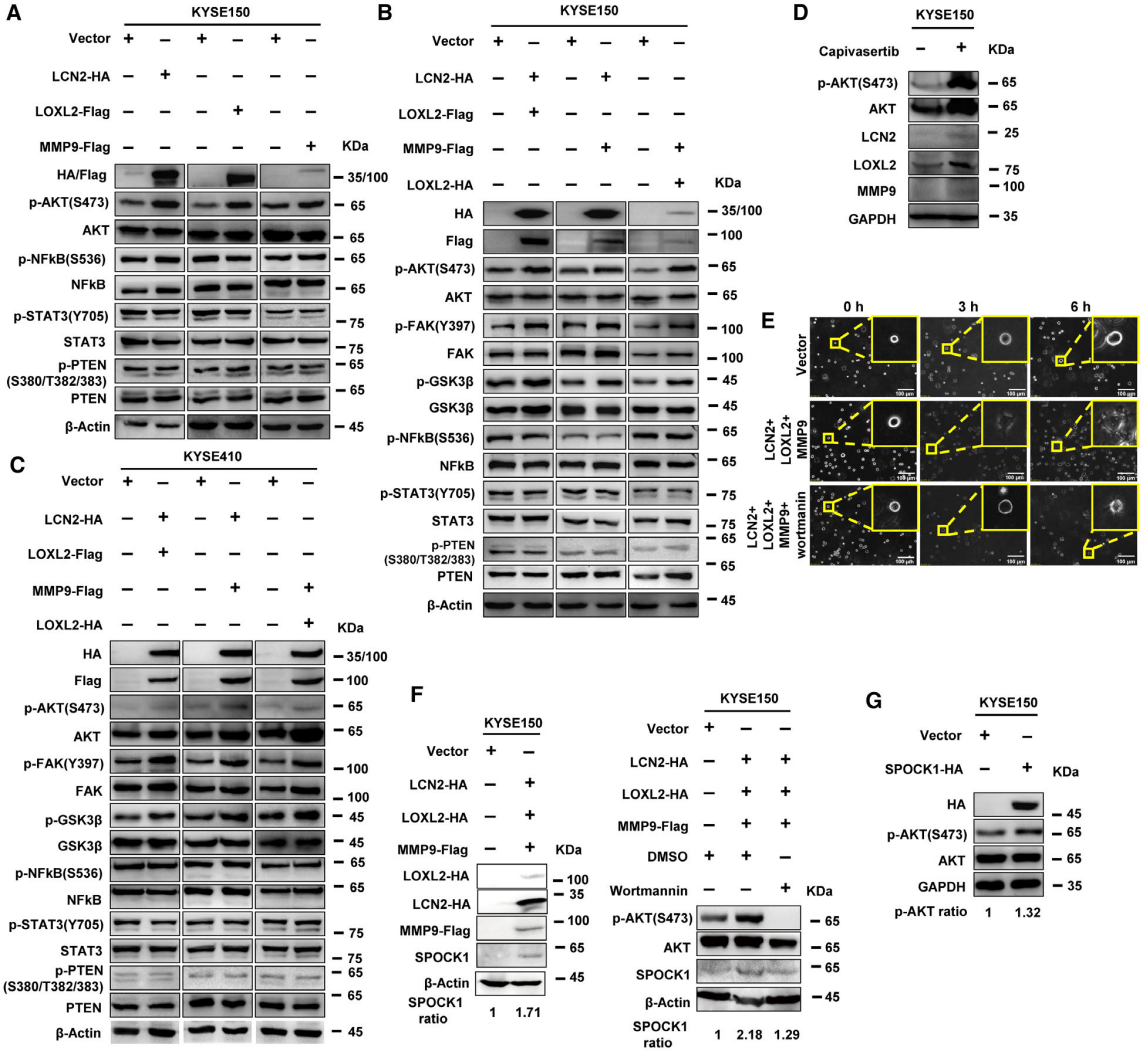
LCN2/LOXL2/MMP9 protein–protein interaction activates the FAK/AKT/GSK3β signalling pathway. (A) After overexpressing LCN2‐HA, LOXL2‐Flag, and MMP9‐Flag, the phosphorylation levels of AKT, NF‐κB, STAT3 and PTEN were detected. In KYSE150 (B) and KYSE410 (C) cells, the phosphorylation levels of AKT, FAK, GSK3β, NF‐κB, STAT3 and PTEN were analysed after co‐transfection of LCN2‐HA/LOXL2‐Flag, LCN2‐HA/MMP9‐Flag, or LOXL2‐HA/MMP9‐Flag. (D) The AKT kinase inhibitor capivasertib was added to KYSE150 cells and the expression of p‐AKT (S473), LCN2, LOXL2 and MMP9 was measured. (E) *LCN2*/*LOXL2*/*MMP9* were overexpressed in KYSE150 cells, and the treated group was treated with the PI3K inhibitor wortmannin. The cells were mixed with Matrigel to construct a 3D culture model, and cell morphological changes and the formation of filopodia were observed at different time points. Scale bars, 20 μm. (F) Alteration of *SPOCK 1* expression was detected after overexpressing LCN2‐HA/LOXL2‐HA/MMP9‐Flag. Wortmannin was added to detect the changes of the phosphorylation levels of AKT and the level of SPOCK1. The relative fold changes of SPOCK1 were quantified by ImageJ software. (G) Alterations of AKT phosphorylation (S473) were detected after overexpression of SPOCK1. Representative images were shown with at least three times in (A–G).

Next, we sought to identify the signalling pathways involving LLM. After *LCN2*, *LOXL2*, and *MMP9* were overexpressed individually, the phosphorylation levels of NF‐κB, STAT3, and PTEN remained unchanged, while the phosphorylation level of AKT increased, indicating that this signalling pathway was activated (Fig. 7A). After co‐overexpression of *LCN2*/*LOXL2*, *LCN2*/*MMP9*, and *LOXL2*/*MMP9*, the phosphorylation levels of NF‐κB, STAT3, and PTEN still remained unchanged, while the phosphorylation levels of FAK, AKT, and GSK3β increased, suggesting that LCN2/LOXL2/MMP9 protein–protein interactions promoted migration and invasion of ESCC cells by activating the FAK/AKT/GSK3β signalling pathway (Fig. 7B). Similar alterations were observed in KYSE410 cells (Fig. 7C). However, co‐overexpression of *LCN2*/*LOXL2*, *LCN2*/*MMP9*, or *LOXL2*/*MMP9* induced no significant change in the level of phosphorylated ERK (Fig. S10).

For further confirmation, pathway activators or inhibitors were applied to confirm the involvement of these signalling pathways. Capivasertib (MCE, HY‐15431), which increases phosphorylation of AKT at both Ser473 and Thr308 [21], was added to KYSE150 cells to mimic activation by the LCN2/LOXL2/MMP9 complex. Compared with the control group, AKT phosphorylation was enhanced by capivasertib, and the expression levels of LCN2, LOXL2, and MMP9 were increased as well (Fig. 7D). Wortmannin is a pan‐inhibitor of PI3K. When cells overexpressing *LCN2*/*LOXL2*/*MMP9* were grown in Matrigel in a 3D model to observe the changes of cell morphology, no obvious changes were observed in the filopodia number or length, except for enlargement of the cell body itself. However, filopodia in the overexpression group began to form at 3 h after transfection. After 6 h, most cells in the overexpressing group had formed filopodia that extended around the cell surface, thus promoting cell spreading, morphological changes, and cell migration. *LCN2*/*LOXL2*/*MMP9*‐overexpressing cells treated with wortmannin had barely extended filopodia after 6 h, and only part of cells began to form the short filamentous pseudopods (Fig. 7E).

The *SPOCK1* gene encodes a matricellular glycoprotein that belongs to a family of novel Ca^2+^‐binding proteoglycans and promotes cell migration in many tumours [22]. Expression of *SPOCK1* was clearly increased after *LCN2*/*LOXL2*/*MMP9* overexpression, which reshaped the ECM, and then promoted the migration and invasion of ESCC cells. Next, the PI3K inhibitor wortmannin was added to cells overexpressing *LCN2*/*LOXL2*/*MMP9*. Wortmannin treatment resulted in inhibition of *SPOCK1* expression, suggesting that the ternary complex of LCN2/LOXL2/MMP9 activated the AKT signalling pathway to promote *SPOCK1* expression (Fig. 7F). Interestingly, overexpression of *SPOCK1* in KYSE150 cells enhanced phosphorylation of AKT, suggesting a positive regulatory feedback loop between SPOCK1 and p‐AKT involving LCN2/LOXL2/MMP9 (Fig. 7G).

## 4 Discussion

Dysregulation of the ECM structure and components is a key event in tumour progression. The ability of cancer cells to migrate and invade is one of the hallmarks of solid metastatic cancer [23]. It is critical to understand how cancer cells interact with their microenvironment to migrate and invade the surrounding tissue, move toward the vasculature (blood/lymphatic vessels), and extravasate to create distant metastases, so that efficient targets for anti‐cancer therapy can be discovered [24].

Numerous studies have shown that LCN2, a secreted protein, is closely related to the malignant progression of tumours. Elevated *LCN2* expression has been observed in various human solid tumours, including breast, colorectal, ovarian, gastric, ovarian, bladder, kidney, and lung cancers, in addition to ESCC [9]. A higher expression level of *LCN2* is usually associated with tumour size, tumour stage, and invasion of carcinoma cells, and indicates a poor prognosis. These characteristics strongly suggest that LCN2 might be a potential biomarker and therapeutic target in malignancies [9]. Previous studies have reported that LCN2 and MMP9 form a heterodimer through disulfide bonding to protect MMP9 enzyme activity, contributing greatly to tumour invasion and metastasis [11, 25, 26]. High levels of monomeric forms of LCN2 and MMP9, and LCN2‐MMP9 heterodimers are secreted into the extracellular space, and their levels seem to correlate with the aggressive behaviour of neoplastic cells in several types of cancer [9]. LOXL2 effectively enhanced MMP9 enzyme activity by elevating MMP9 expression [27]. LOXL2 catalyses the cross‐linkage of extracellular collagen to change the stiffness of the ECM, facilitating the motility of cancer cells [27, 28]. MMP9 plays a core role in the degradation of the extracellular matrix and basement membrane of cancers to contribute to tumour invasion and metastasis [29, 30].

Considering the crosstalk of functional roles of *LCN2*, *MMP9* and *LOXL2* in tumour progression, we hypothesized that an LCN2/LOXL2/MMP9 ternary complex might exist in oesophageal cancer and play a synergistic role in biological function. First, we used mass spectroscopy to show that LCN2 directly interacted with LOXL2. A LCN2/LOXL2 PPI network suggested that a physical interaction might occur between LCN2 and LOXL2, which might also involve other interacting proteins, such as MMP9. Our results showed that an LCN2/LOXL2/MMP9 ternary complex was formed in oesophageal cancer, with a distinctive intracellular and extracellular interaction pattern. The SRCR structure is a key domain through which LOXL2 interacts with both LCN2 and MMP9 to play an important biological role in oesophageal cancer. In recent years, the N‐terminal SRCR repeats of the LOX gene family have been found to play an important functional role [11]. Similarly, the SRCR domain in LOXL3, rather than the C‐terminal oxidase catalytic domain, represents the major deacetylase/deacetyliminase activity in the modification of STAT3 [31].

Based on LCN2/LOXL2, LCN2/MMP9 and LOXL2/MMP9 protein–protein interactions, we further elucidated the molecular and cellular mechanisms underlying the LCN2/LOXL2/MMP9 ternary complex that promoted migration and invasion of oesophageal cancer cells, playing a synergistic role. Zymography showed that an elevated LCN2/LOXL2/MMP9 ternary complex was presented, especially in the case of enhanced expression of MMP9. MMP9 has three fibronectin type II homologous repeat domains that bind to gelatin with high affinity, making gelatin the major substrate for MMP9 [32, 33]. Degradation of fluorescent fibronectin (FN) experiments revealed that co‐expression of *LCN2*/*LOXL2*, *LCN2*/*MMP9*, or *LOXL2*/*MMP9* conferred a greater ability to degrade FN. FN is a major non‐collagen glycoprotein in the extracellular matrix and basement membrane, which adheres fibrin and collagen [34]. A previous study found that a high stromal FN content facilitated tumour cell metastasis by promoting morphological change and improving the motility and migratory ability of ESCC cells [35]. Therefore, the LCN2/LOXL2/MMP9 ternary complex can promote the invasion of oesophageal cancer cells by elevating the expression of MMPs to degrade gelatin and fibronectin. Considering that growth of tumour cells *in vivo* occurs in a three‐dimensional environment, 3D cell culture showed that the LCN2/LOXL2/MMP9 ternary complex degraded the extracellular matrix by promoting the formation and extension of filopodia, thereby enhancing the invasion of tumour cells. Matrigel® is widely used in cancer cell 3D cell culture models and contains proteins commonly found in the basement membrane of epithelial structures, such as laminin, as well as type IV collagen and heparan sulfate proteoglycan [36, 37]. MMP9 was able to degrade the laminin and type IV collagen in Matrigel. Therefore, we concluded that the LCN2/LOXL2/MMP9 ternary complex promoted the invasion of cancer cells by degrading laminin and type IV collagen.

Aside from degradation of the extracellular matrix, the mobility of tumour cells is also an important factor that contributes to migration and invasion. Actin is a highly conserved protein that participates in various types of cell movement and is universally expressed in all eukaryotic cells. Polymerization of individual actin filaments into organized parallel bundles occurs in filopodia, which are slender protrusions that can extend far beyond the cell edge and reach and sense distant targets [38]. PFN1 is an abundant actin‐binding protein that promotes nucleotide exchange of actin and converts ADP/G‐actin to ATP/G‐actin [20]. The PFN1‐ATP/G‐actin complex interacts with the fast‐growing end of F‐actin to increase ATP/G‐actin for growing actin filaments. Thus, profilin 1 is a central mediator of actin microfilament and microtubule dynamics [20]. Cells overexpressing *LCN2*/*LOXL2*, *LCN2*/*MMP9*, *LOXL2*/*MMP9*, or LLM exhibited a disordered microfilament skeleton, increased filopodia, and increased expression of *profilin 1* distributed in the cell interior and the leading edge of the cell. These results suggested that LCN2/LOXL2/MMP9 enhanced cell migration by promoting microfilament rearrangement and enhancing *profilin 1* expression. The cytoskeleton, with its regulatory and structural proteins, has emerged as a novel and highly effective target to be exploited for specific anti‐metastatic drugs [39]. Although this appears contradictory when considering their individual role in ECM remodelling, LCN2/MMP9 mainly contribute to matrix degradation, while LOXL2 strengthens ECM stiffness by catalysing the cross‐linking of collagen and elastin. Nevertheless, we provide evidence that LCN2/LOXL2, LCN2/MMP9, LOXL2/MMP9, and LLM affect tumour growth and progression in a synergistic manner. We presumed that ECM degradation opens a path, and ECM stiffness favours the assembly of filopodia and the invadosome, both of which contribute to cancer cell migration and invasion.

Iron (Fe) plays a vital role in various biological processes. Cancer cells show a high rate of Fe metabolism and therefore require more Fe than normal cells to support high proliferation rates. The evidence indicates that tumour cells are very sensitive to iron deficiency, much more sensitive than normal cells [40]. LCN2 is an important ferritin carrier that increases the iron level in cells to promote the progression of malignant behaviour [41]. Fe chelating agents have been used to treat a variety of diseases, such as leukaemia, neuroblastoma and breast cancer [42]. Among them, DFOM has been extensively studied and found to have anti‐tumour effects by reducing iron overload [43, 44]. In this study, we analysed the effect of DFOM in ESCC tumour progression by targeting LCN2, and the LCN2/LOXL2/MMP9 complex as well. We showed that DFOM inhibited the migration and invasion of oesophageal cancer cells through multiple mechanisms, including reducing the expression of *LCN2* and LLM, suppressing MMP9 enzyme activity, inhibiting cytoskeletal rearrangement, and thus restoring ECM remodelling. Furthermore, our *in vivo* experiments showed that DFOM inhibited ESCC tumour growth.

**Fig. 8:**
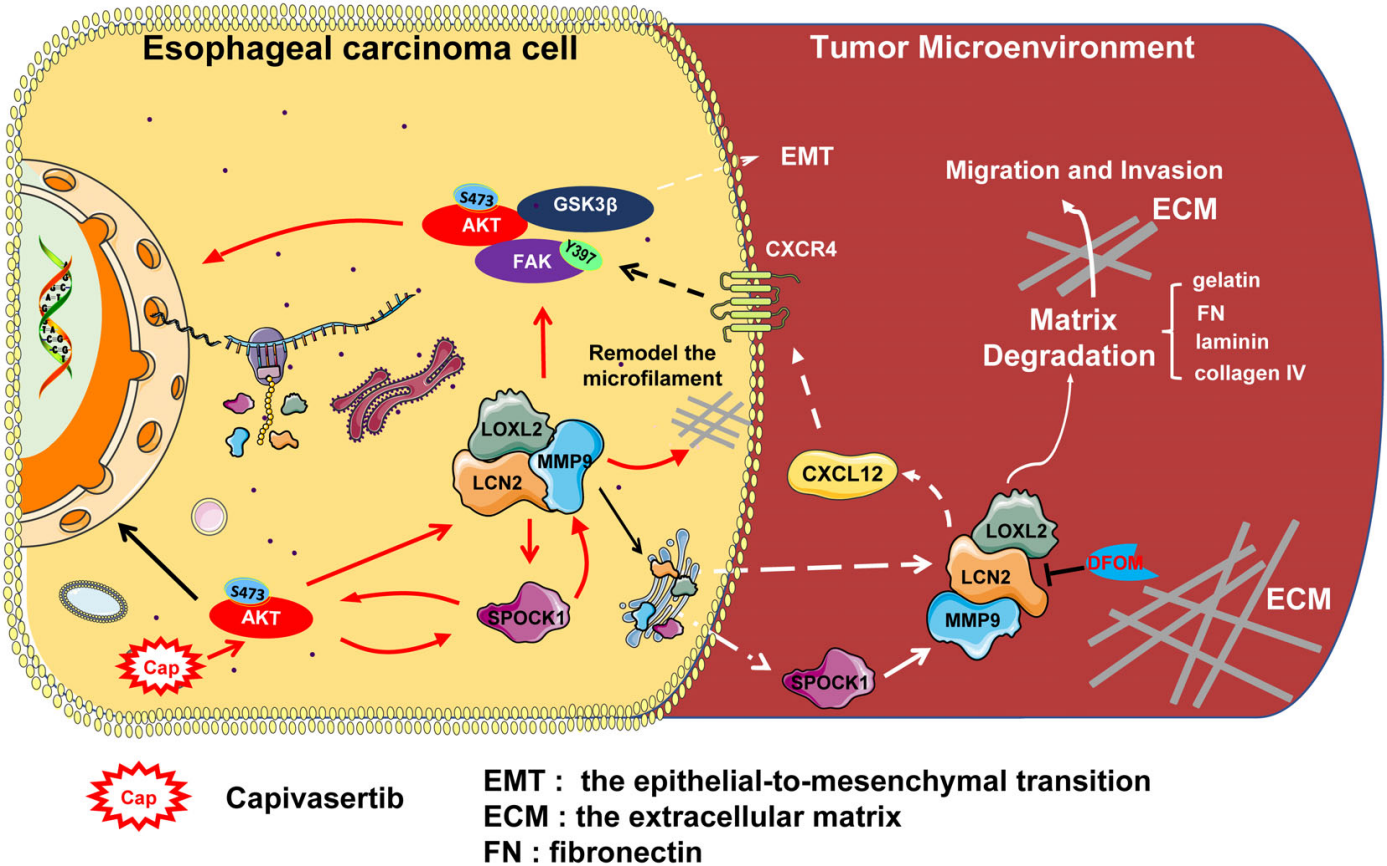
A model for LCN2/LOXL2/MMP9 complex enhancement of migration and invasion through the FAK/AKT/GSK3β signalling pathway and extracellular matrix degradation.

Leng et al. [45] found that AKT signalling upregulated *LCN2* expression and promoted breast cancer invasion and migration. It has been reported that LCN2 activates the PI3K/AKT pathway in smooth muscle cells [46]. In endothelial cells, LOXL2 activates AKT and FAK signalling, which participate in the regulation of EMT [47]. Zheng et al. [48] found that MMP9 promoted the occurrence of lung cancer through the PI3K/AKT signalling pathway. In this study, we found that the LCN2/LOXL2/MMP9 ternary complex activated the FAK/AKT/GSK3β signalling pathway to promote expression of *SPOCK1*. *SPOCK1* is closely related to cell proliferation, adhesion, and metastasis, and it is highly expressed in a variety of cancers, including ESCC, liver, gallbladder, colon, and prostate cancer [49]. Liu et al. [50] found that SPOCK1 upregulated the expression and activity of *MMP9*, causing remodelling of the ECM and promoting tumour cell migration and invasion. Therefore, LCN2/LOXL2/MMP9 activated the FAK/AKT/GSK3β signalling pathway to enhance *SPOCK1* expression and remodel the ECM, thus promoting the migration and invasion of oesophageal cancer cells. Moreover, degradation of the ECM was inhibited after adding the PI3K inhibitor wortmannin, suggesting that inhibition of AKT phosphorylation at Ser473 inhibited the migration and invasion of oesophageal cancer. Therefore, AKT (Ser473) phosphorylation could be a potential therapeutic target for oesophageal cancer. Taken together, we propose a schematic model in which the LCN2/LOXL2/MMP9 complex promotes the migration and invasion of cancer cells through intracellular and extracellular means (Fig. 8).

## 5 Conclusions

In conclusion, we found that an LCN2/LOXL2/MMP9 ternary complex activated the FAK/AKT/GSK3β signalling pathway, degraded extracellular matrix components, such as FN and collagen IV, remodelled the cytoskeleton, and enhanced the expression of *profilin 1* and *SPOCK1* to promote the migration and invasion of oesophageal cancer cells, all of which ultimately led to malignant progression of oesophageal cancer. In contrast, DFOM inhibited the progression of oesophageal cancer. *LCN2*, *LOXL2*, and *MMP9* might serve as promising therapeutic targets in solid tumours, including ESCC.

## Conflict of interest

The authors declare no conflict of interest.

## Author contributions

Conceptualization, BW, EL, and ZD; Methodology & investigation, QX, MC, X Zhou, WB, X Zheng, LL, YZ, JD, and ZW; Supervision, LX; Data curation, HZ and SW; Writing ‐ original draft, QX; Writing ‐ review & editing: BW, EL, and ZD; Funding acquisition, BW and ZD. All authors read and approved the final manuscript.

### Peer review

The peer review history for this article is available at https://www.webofscience.com/api/gateway/wos/peer‐review/10.1002/1878‐0261.13529.

## Supporting information


**Fig. S1.** Prediction of protein–protein interactions and construction of a cell model. (A) The mass spectrum of the VPLQQNFQDNQFQGK proteolysis product of LCN2 protein from the LOXL2 interactome. (B) Construction of protein–protein interaction networks using LCN2 and LOXL2 as seed proteins. Circles represent protein molecules, and links represent protein–protein interactions. (C) Pearson expression correlation between LCN2, LOXL2 and MMP9. (D) Endogenous expression level of LCN2 and LOXL2 protein in oesophageal cancer cell lines. (E) Successful expression of LCN2‐HA/LOXL2‐Flag, LCN2‐HA/MMP9‐Flag, and LOXL2‐HA/MMP9‐Flag in the KYSE410 and KYSE150 oesophageal cancer cell lines following co‐transfection.

**Fig. S2.** Immunohistochemistry detection of LCN2, MMP9 and LOXL2 in 20 pairs of ESCC clinical sample. An immunoreactive score was calculated by multiplying the percentage of positive cells and the staining intensity for normal and cancerous oesophageal epithelial tissue, respectively. The difference of scores between normal and tumour was calculated by Mann–Whitney test.

**Fig. S3.** The endogenous interactions between LCN2/LOXL2, LOXL2/MMP9 were detected by immunofluorescence in two ESCC cell lines.

**Fig. S4.** Effect of LCN2/LOXL2/MMP9 protein–protein interaction on the function of ESCC cells. LCN2‐HA/LOXL2‐Flag, LCN2‐HA/MMP9‐Flag, or LOXL2‐HA/MMP9‐Flag were co‐transfected into KYSE410 cells. Wound‐healing (A) and migration assays (B) were used to measure cell migration. (C) Transwell with Matrigel‐coated membranes were used to study the invasive capacity of ESCC cells. (D) In KYSE410 cells, the effects of LCN2, LCN2/LOXL2 and LCN2/LOXL2/MMP9 on migration (left panel) and invasion (right panel) were characterized. Effects on the proliferation of ESCC cells were detected by colony‐forming assay (E). In KYSE410 and KYSE150 cells, MTS assays were performed to detect the proliferation of ESCC cells after the overexpression of LCN2‐HA/LOXL2‐Flag, LCN2‐HA/MMP9‐Flag, or LOXL2‐HA/MMP9‐Flag, respectively (F‐G). (H) Flow cytometry was used to detect changes in the cell cycle.

**Fig. S5.** 3D Reconstruction of confocal Z stack demonstration of truncated LOXL2 co‐localization with LCN2 and MMP9. D4‐HA/LCN2, D4‐HA/MMP9 were overexpressed in KYSE150 cells, and different levels of the sample were scanned using the LSM800 Z‐stack function to observe the colocalization of D4‐HA with LCN2 (A) and MMP9 (B). Pearson correlation coefficients for co‐localization are shown.

**Fig. S6.** The analysis of DFOM IC50 by MTS assays with 0, 5, 10, 25, 50, 100 and 200 μM treatment.

**Fig. S7.** Extracellular matrix degradation was observed by Z‐axis analysis. Fluorescent matrix degradation assay was used to observe the degradation of ECM. (A) Vector control. (B) LCN2 + LOXL2 overexpression. (C) LCN2 + MMP9 overexpression. (D) LOXL2 + MMP9 overexpression. The LSM800 Z‐stack function was applied to scan different layers of the sample. FN488 fluorescent matrix was shown in green, F‐actin was stained in red, and blue colour represented nuclei stained by DAPI. Scale bar, 20 μm.

**Fig. S8.** (A‐C) The quantifications of the length of filopodia for Fig. 5 C, D and F, respectively. It shows the overexpression of LCN2, LOXL2 and MMP9 increased the length of filopodia, while were inhibited by DFOM.

**Fig. S9.** Effect of truncated LOXL2 and full‐length LOXL2 on the growth of filopodia. (A) The full‐length and truncated LOXL2 plasmid was transfected into KYSE150 cells. Overexpressing cells were suspended in Matrigel to construct a 3D culture model. Degradation of the matrix and formation of cell filopodia were observed at different times. (B) The qualifications of the length of filopodia in truncated LOXL2 and full length LOXL2 overexpression cells.

**Fig. S10.** Variation of ERK phosphorylation level. LCN2, LOXL2 and MMP9 were transfected individually (A), or LCN2/LOXL2, LCN2/MMP9 and LOXL2/MMP9 were co‐transfected in combination (B), to detect the phosphorylation level of ERK.

**Table S1.** List of antibodies in this study.

**Table S2.** Peptides used to Identify LCN2 Protein by MS.

## Data Availability

All data generated in this study have been included in the article and additional files.
