# Supplementary figures and images for: A protein complex of LCN2, LOXL2 and MMP9 facilitates tumour metastasis in oesophageal cancer
Source: Mol Oncol. 2023 Oct 4;17(11):2451–71. doi: 10.1002/1878-0261.13529 (PMC10620126; doi:10.1002/1878-0261.13529)

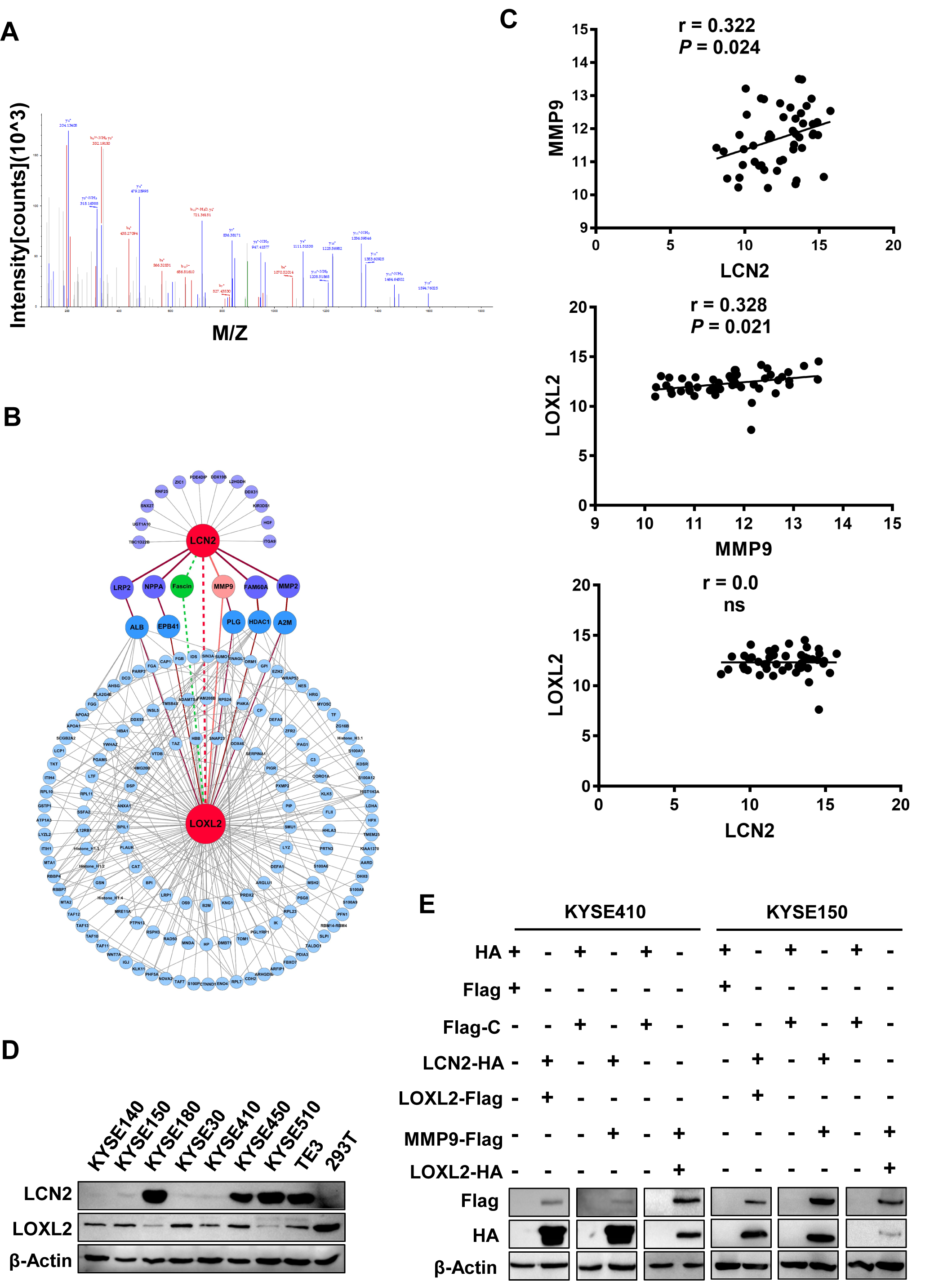

Supplement: Supplementary file 1 — Fig. S1. Prediction of protein–protein interactions and construction of a cell model. (A) The mass spectrum of the VPLQQNFQDNQFQGK proteolysis product of LCN2 protein from the LOXL2 interactome. (B) Construction of protein–protein interaction networks using LCN2 and LOXL2 as seed proteins. Circles represent protein molecules, and links represent protein–protein interactions. (C) Pearson expression correlation between LCN2, LOXL2 and MMP9. (D) Endogenous expression level of LCN2 and LOXL2 protein in oesophageal cancer cell lines. (E) Successful expression of LCN2‐HA/LOXL2‐Flag, LCN2‐HA/MMP9‐Flag, and LOXL2‐HA/MMP9‐Flag in the KYSE410 and KYSE150 oesophageal cancer cell lines following co‐transfection. [file MOL2-17-2451-s008.tif]

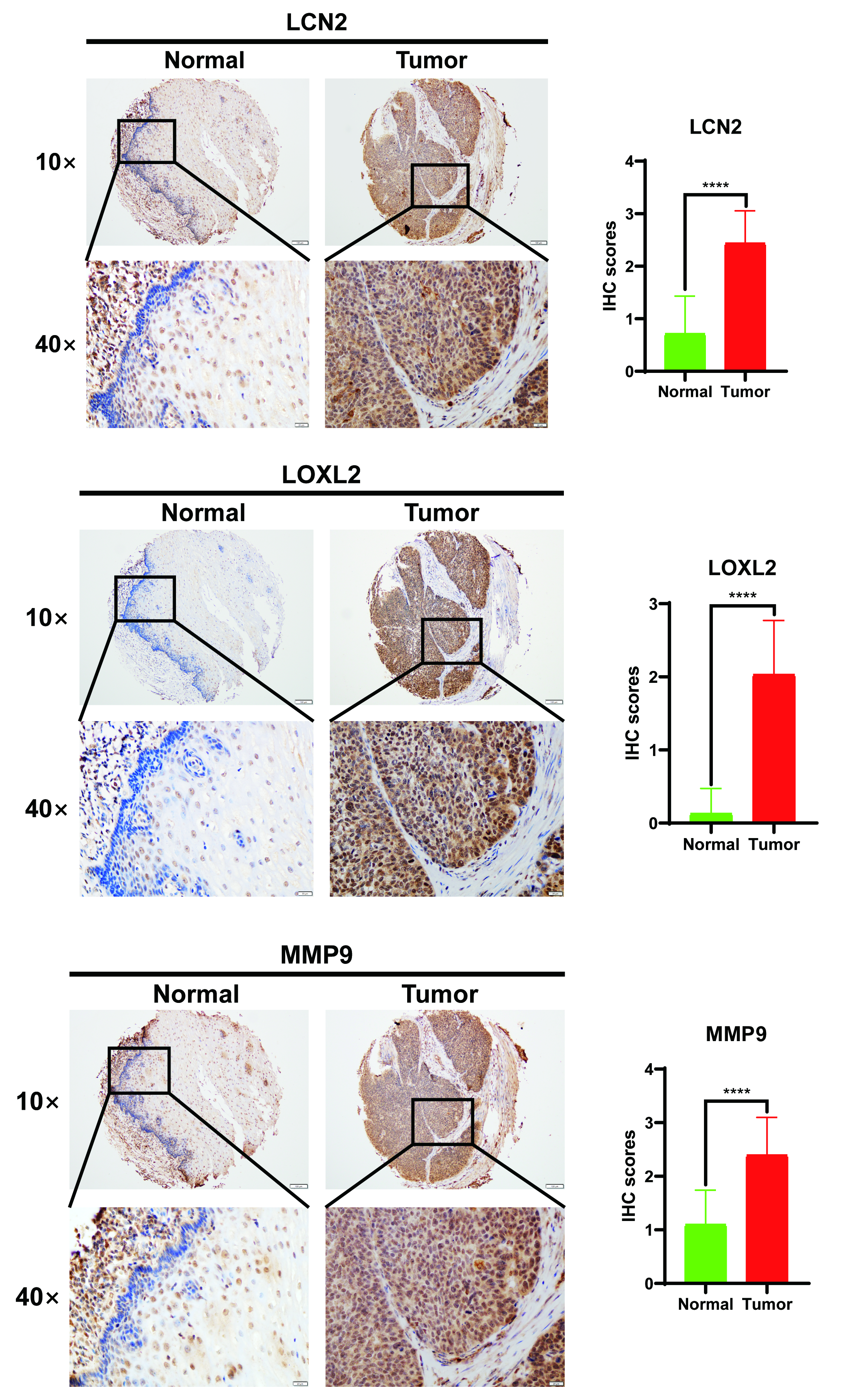

Supplement: Supplementary file 2 — Fig. S2. Immunohistochemistry detection of LCN2, MMP9 and LOXL2 in 20 pairs of ESCC clinical sample. An immunoreactive score was calculated by multiplying the percentage of positive cells and the staining intensity for normal and cancerous oesophageal epithelial tissue, respectively. The difference of scores between normal and tumour was calculated by Mann–Whitney test. [file MOL2-17-2451-s011.tif]

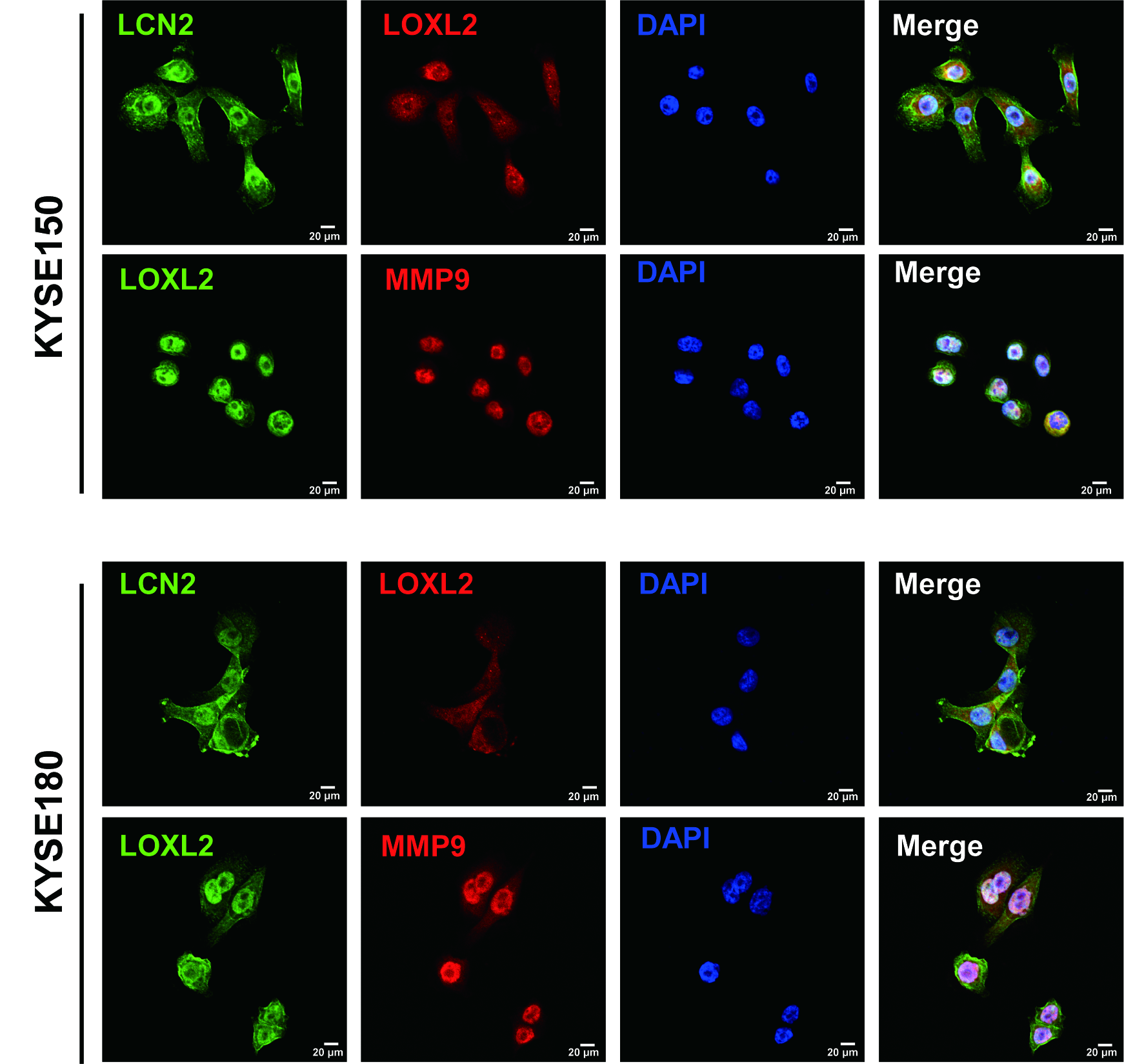

Supplement: Supplementary file 3 — Fig. S3. The endogenous interactions between LCN2/LOXL2, LOXL2/MMP9 were detected by immunofluorescence in two ESCC cell lines. [file MOL2-17-2451-s004.tif]

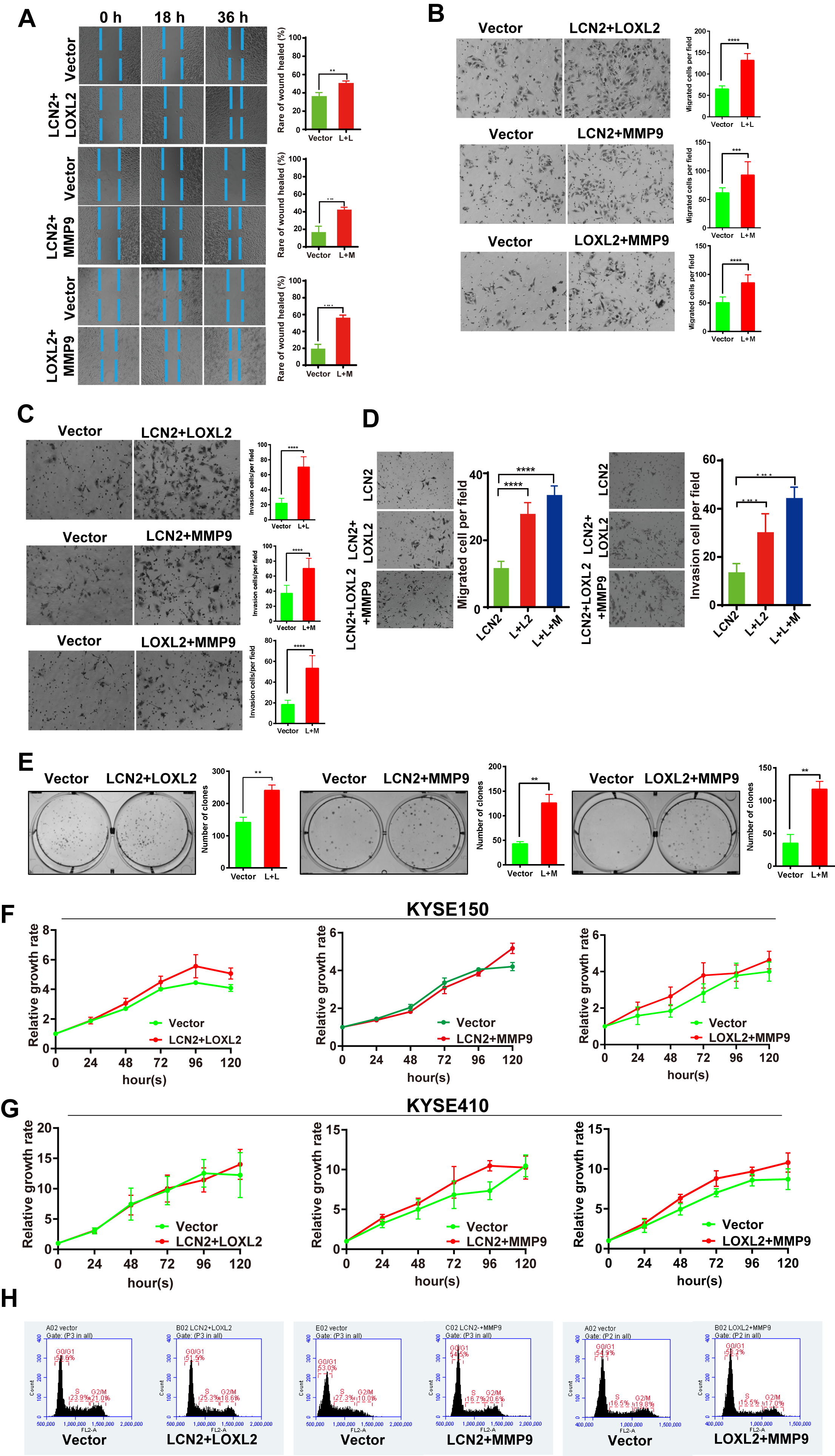

Supplement: Supplementary file 4 — Fig. S4. Effect of LCN2/LOXL2/MMP9 protein–protein interaction on the function of ESCC cells. LCN2‐HA/LOXL2‐Flag, LCN2‐HA/MMP9‐Flag, or LOXL2‐HA/MMP9‐Flag were co‐transfected into KYSE410 cells. Wound‐healing (A) and migration assays (B) were used to measure cell migration. (C) Transwell with Matrigel‐coated membranes were used to study the invasive capacity of ESCC cells. (D) In KYSE410 cells, the effects of LCN2, LCN2/LOXL2 and LCN2/LOXL2/MMP9 on migration (left panel) and invasion (right panel) were characterized. Effects on the proliferation of ESCC cells were detected by colony‐forming assay (E). In KYSE410 and KYSE150 cells, MTS assays were performed to detect the proliferation of ESCC cells after the overexpression of LCN2‐HA/LOXL2‐Flag, LCN2‐HA/MMP9‐Flag, or LOXL2‐HA/MMP9‐Flag, respectively (F‐G). (H) Flow cytometry was used to detect changes in the cell cycle. [file MOL2-17-2451-s012.tif]

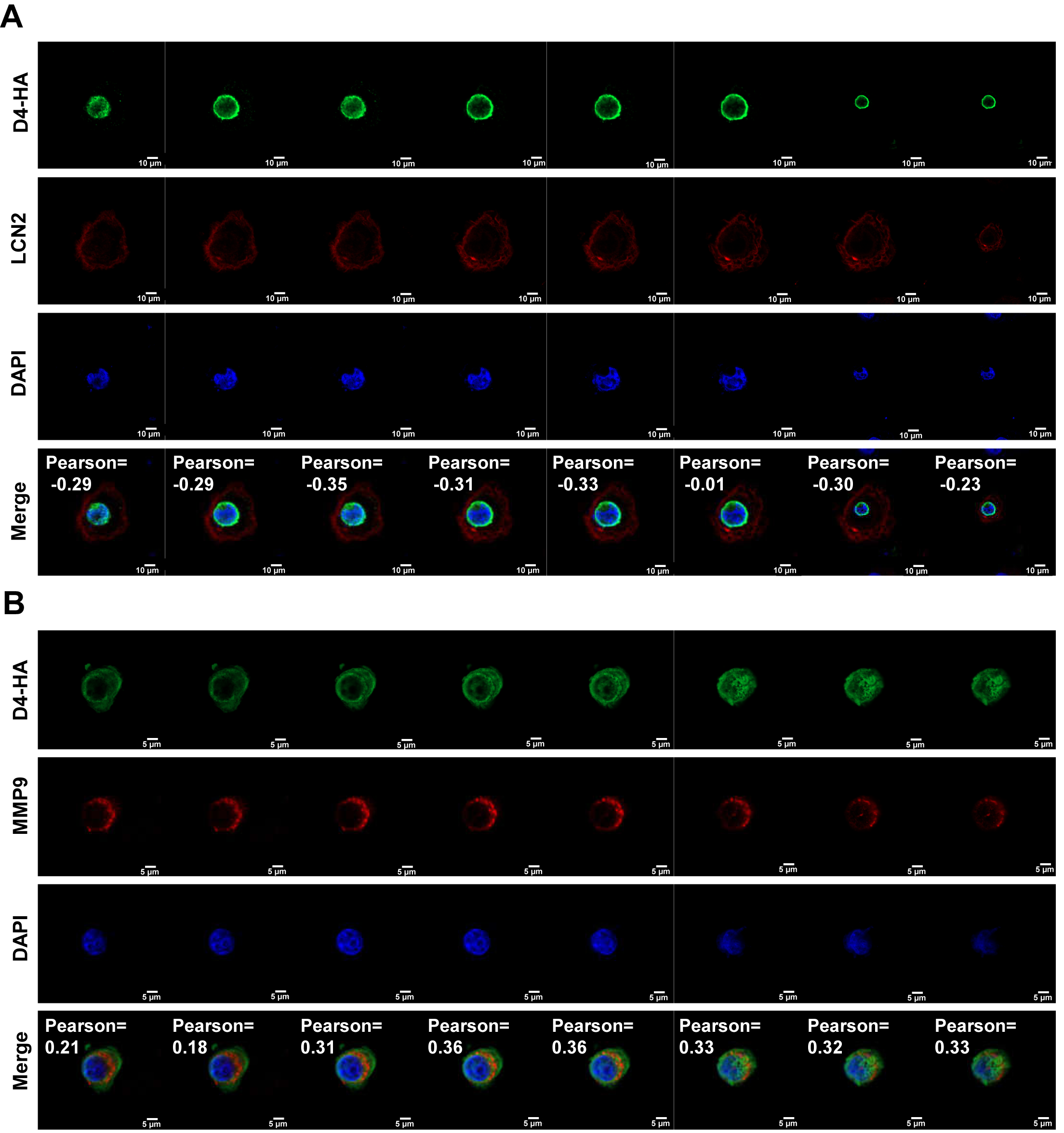

Supplement: Supplementary file 5 — Fig. S5. 3D Reconstruction of confocal Z stack demonstration of truncated LOXL2 co‐localization with LCN2 and MMP9. D4‐HA/LCN2, D4‐HA/MMP9 were overexpressed in KYSE150 cells, and different levels of the sample were scanned using the LSM800 Z‐stack function to observe the colocalization of D4‐HA with LCN2 (A) and MMP9 (B). Pearson correlation coefficients for co‐localization are shown. [file MOL2-17-2451-s001.tif]

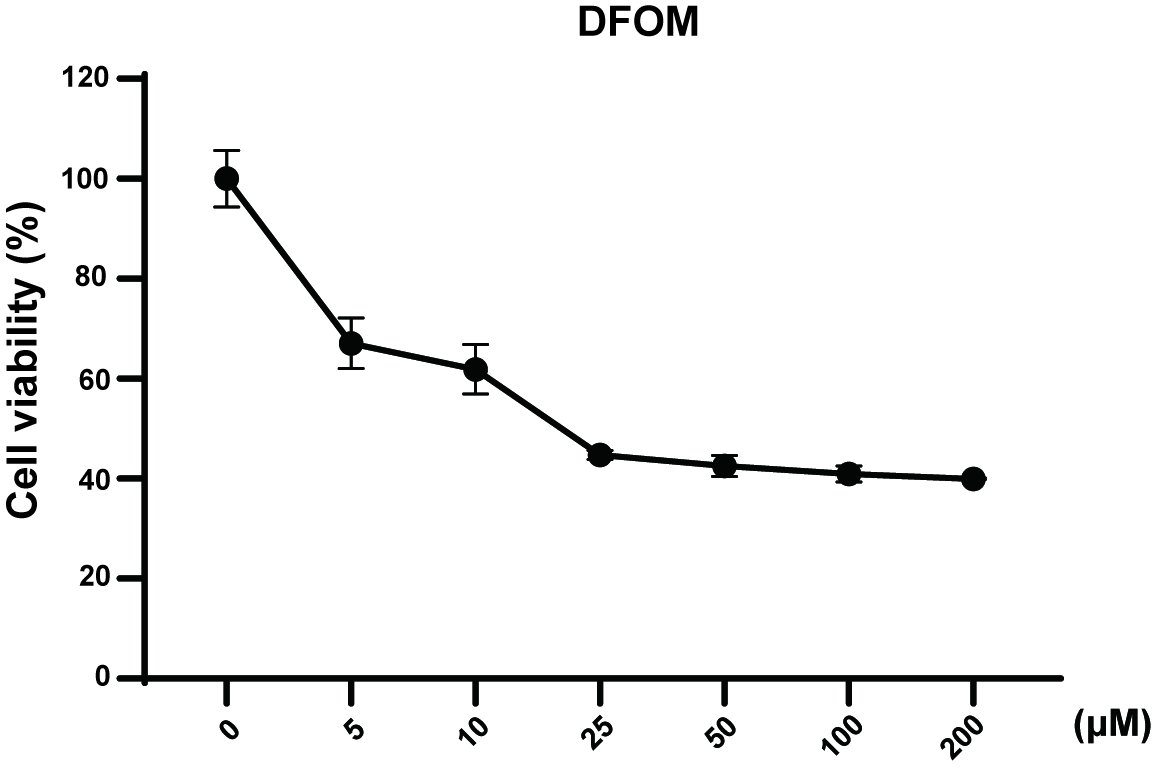

Supplement: Supplementary file 6 — Fig. S6. The analysis of DFOM IC50 by MTS assays with 0, 5, 10, 25, 50, 100 and 200 μM treatment. [file MOL2-17-2451-s002.tif]

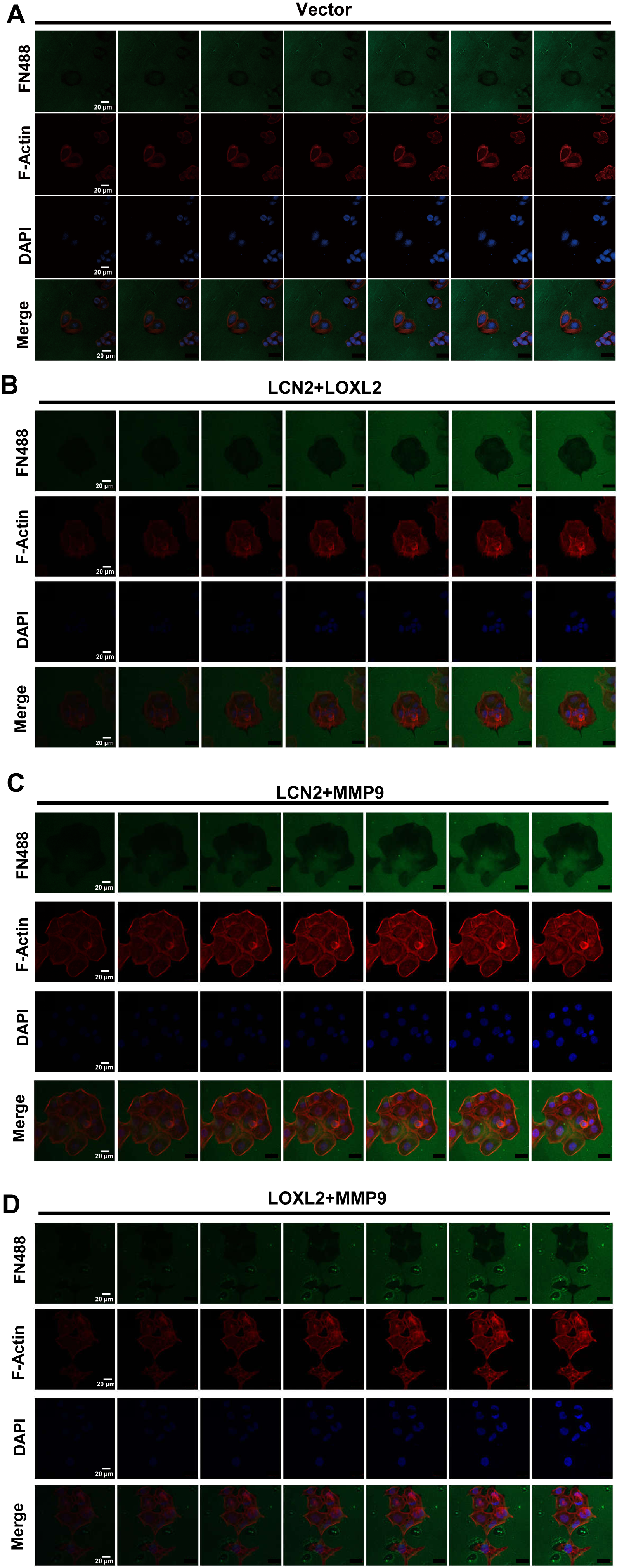

Supplement: Supplementary file 7 — Fig. S7. Extracellular matrix degradation was observed by Z‐axis analysis. Fluorescent matrix degradation assay was used to observe the degradation of ECM. (A) Vector control. (B) LCN2 + LOXL2 overexpression. (C) LCN2 + MMP9 overexpression. (D) LOXL2 + MMP9 overexpression. The LSM800 Z‐stack function was applied to scan different layers of the sample. FN488 fluorescent matrix was shown in green, F‐actin was stained in red, and blue colour represented nuclei stained by DAPI. Scale bar, 20 μm. [file MOL2-17-2451-s006.tif]

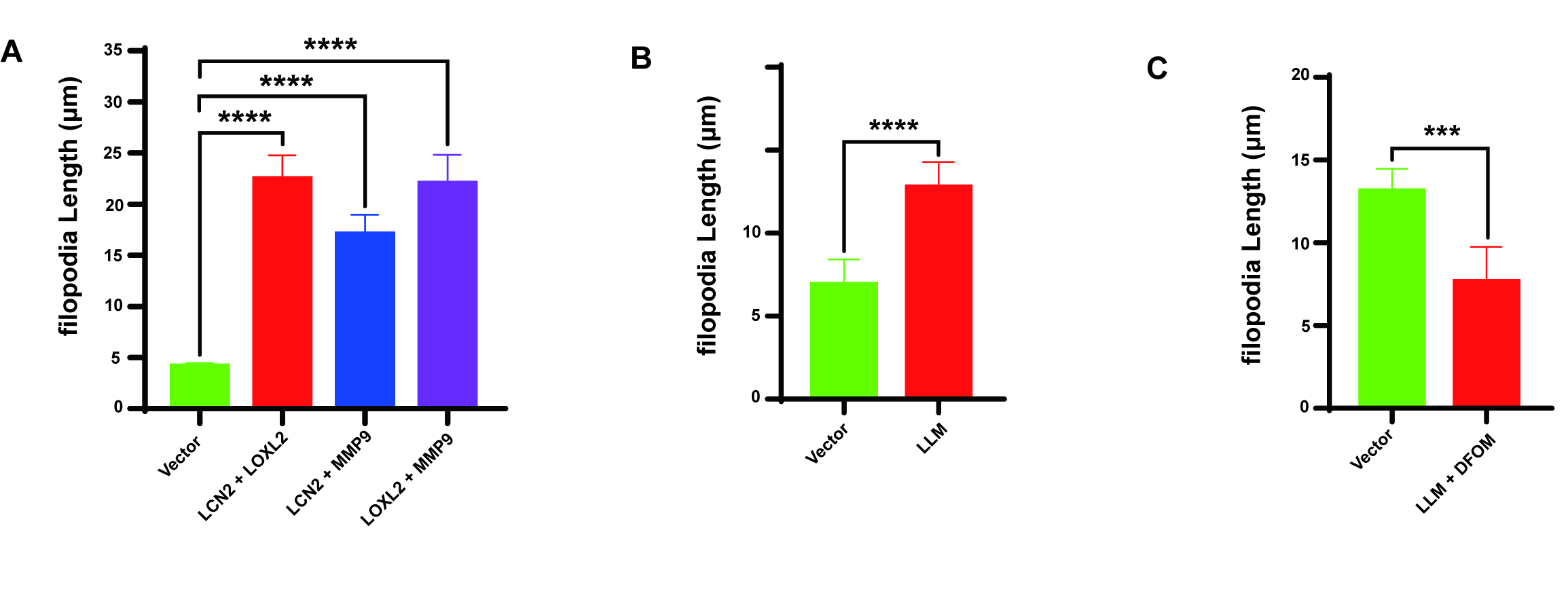

Supplement: Supplementary file 8 — Fig. S8. (A‐C) The quantifications of the length of filopodia for Fig. 5 C, D and F, respectively. It shows the overexpression of LCN2, LOXL2 and MMP9 increased the length of filopodia, while were inhibited by DFOM. [file MOL2-17-2451-s003.tif]

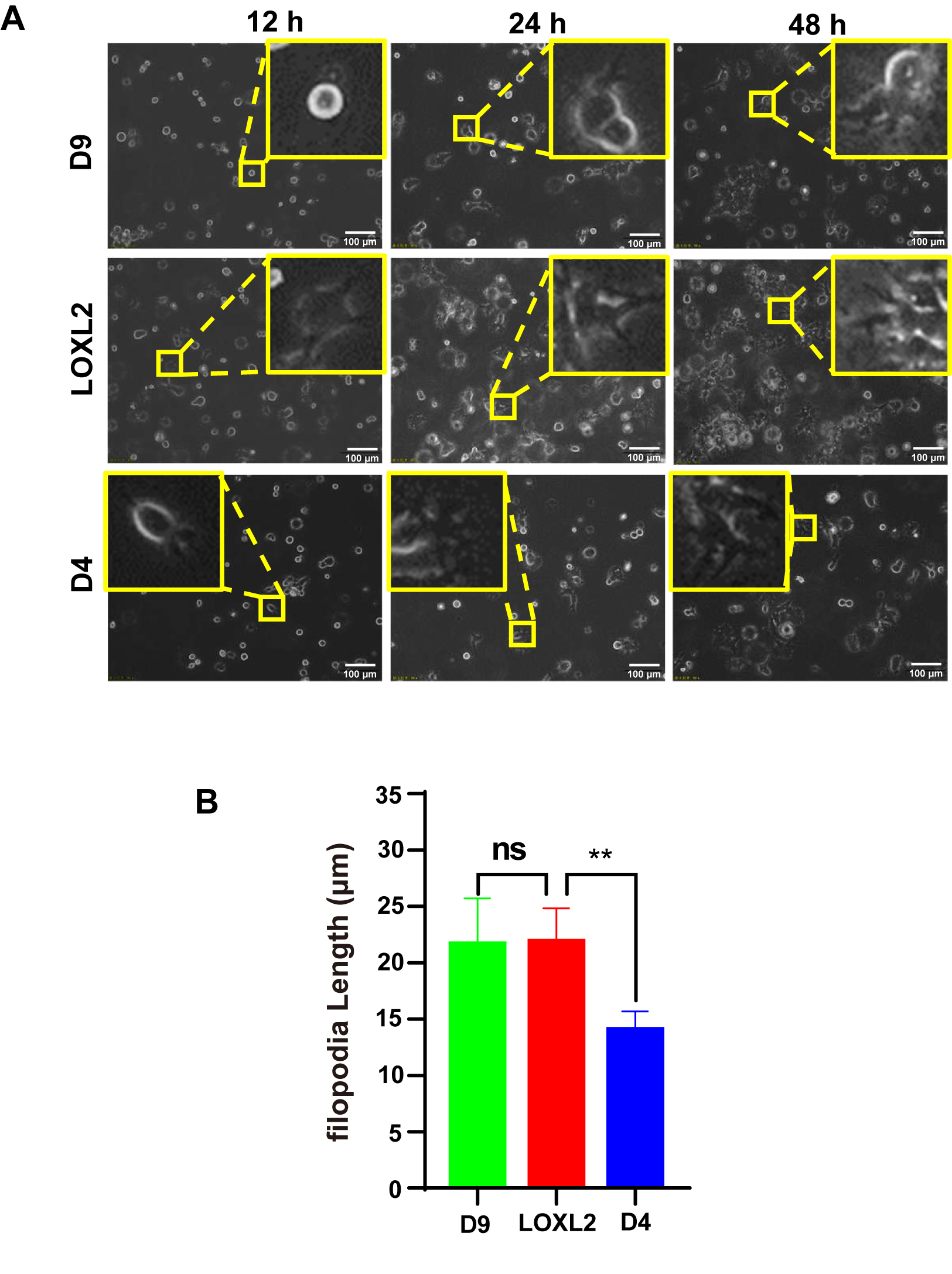

Supplement: Supplementary file 9 — Fig. S9. Effect of truncated LOXL2 and full‐length LOXL2 on the growth of filopodia. (A) The full‐length and truncated LOXL2 plasmid was transfected into KYSE150 cells. Overexpressing cells were suspended in Matrigel to construct a 3D culture model. Degradation of the matrix and formation of cell filopodia were observed at different times. (B) The qualifications of the length of filopodia in truncated LOXL2 and full length LOXL2 overexpression cells. [file MOL2-17-2451-s010.tif]

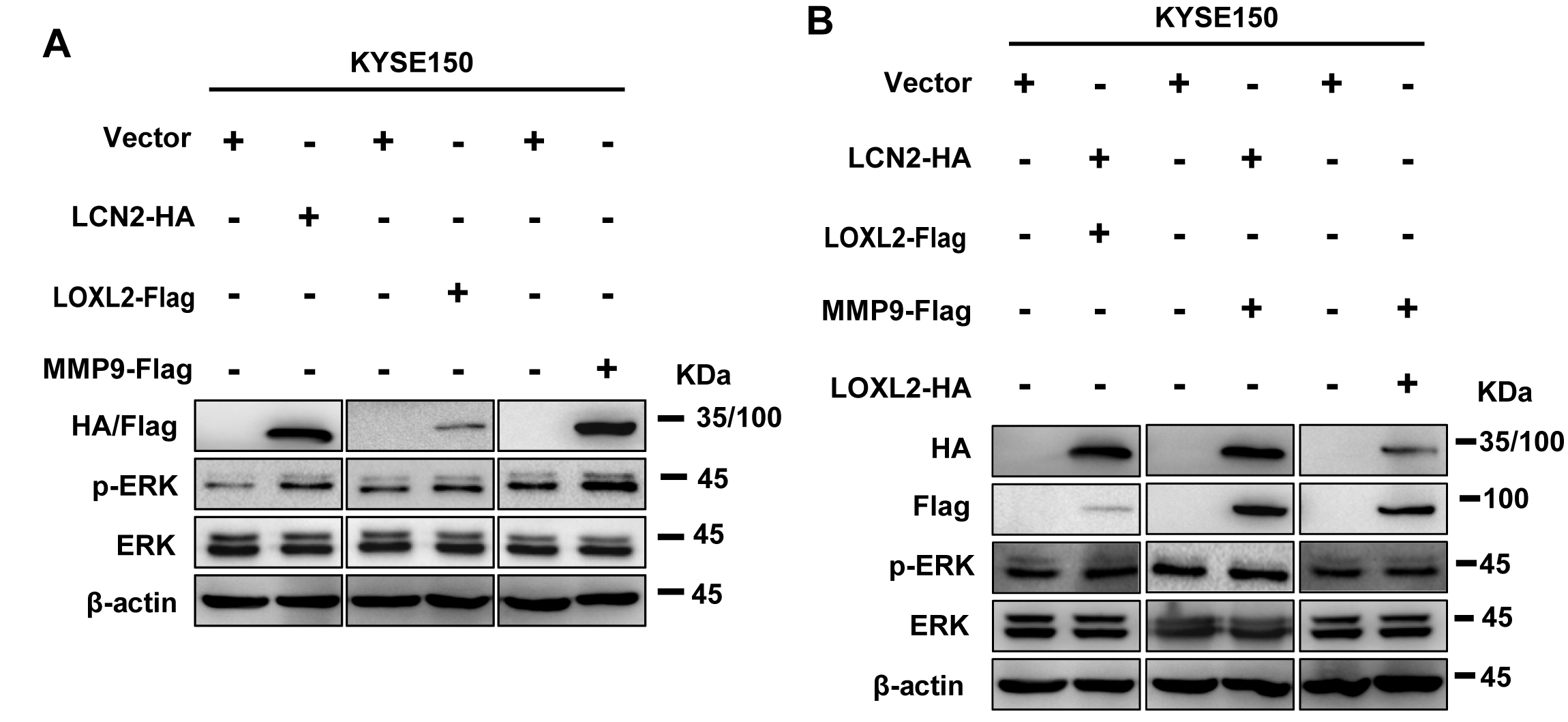

Supplement: Supplementary file 10 — Fig. S10. Variation of ERK phosphorylation level. LCN2, LOXL2 and MMP9 were transfected individually (A), or LCN2/LOXL2, LCN2/MMP9 and LOXL2/MMP9 were co‐transfected in combination (B), to detect the phosphorylation level of ERK. [file MOL2-17-2451-s007.tif]
