# Supplementary material for: A protein complex of LCN2, LOXL2 and MMP9 facilitates tumour metastasis in oesophageal cancer
Source: Mol Oncol. 2023 Oct 4;17(11):2451–71. doi: 10.1002/1878-0261.13529 (PMC10620126; doi:10.1002/1878-0261.13529)
Supplement: Supplementary file 11 — Table S1. List of antibodies in this study. [file MOL2-17-2451-s005.docx]

| **Supplementary Table 1. List of antibodies in this study** | | | |
| --- | --- | --- | --- |
| **Company** | **Antibody** | **Cat. number** | **Dilution (WB)** |
| CST | rabbit mAb anti-phospho-FAK (Tyr397) (D20B1) | 8556T | 1:1000 |
|  | rabbit mAb anti-FAK (D2R2E) | 13009T | 1:1000 |
|  | rabbit mAb anti-phospho-GSK-3β (Ser9) (D85E12) | 5558T | 1:1000 |
|  | rabbit mAb anti-GSK-3β (D5C5Z) | 12456T | 1:1000 |
|  | rabbit mAb anti-phospho-NF-κB p65 (Ser536) (93H1) | 3033T | 1:1000 |
|  | rabbit mAb anti-NF-κB p65 (D14E12) | 8242T | 1:1000 |
|  | rabbit mAb anti-phospho-Stat3 (Tyr705) (D3A7) | 9145S | 1:1000 |
|  | rabbit mAb anti-Stat3 (D3Z2G) | 12640S | 1:1000 |
|  | rabbit mAb anti-phospho-PTEN (Ser380/Thr382/383) (44A7) | 9549T | 1:1000 |
|  | rabbit mAb anti-PTEN (D4.3) | 9188T | 1:1000 |
|  | rabbit mAb anti-phospho-Akt (Ser473) (D9E) | 4060T | 1:2000 |
|  | rabbit mAb anti-Akt (pan) (C67E7) | 4691T | 1:2000 |
|  | rabbit mAb anti-LCN2 (D4M8L) | D4M8L | 1:1000 |
|  | rabbit mAb anti-MMP-9 (D6O3H) | 13667S | 1:1000 |
|  | goat anti-rabbit | 7074P2 | 1:1000 |
| NOVUS | rabbit anti-LOXL2 | NBP1-32954 | 1:1000 |
| Santa Cruz | mouse anti- LOXL2 | Sc-293427 | 1:50 |
|  | mouse mAb anti-HA-probe (F-7) | sc-7392 | 1:1000 |
|  | rabbit anti-ERK1 | sc-94 | 1:1000 |
|  | mouse mAb anti-p-ERK | sc-7383 | 1:1000 |
| Thermo | mouse mAB anti-DYKDDDDK Tag | MA1-9187 | 1:1000 |
|  | mouse mAb anti-β-actin | MA5-15739 | 1:5000 |
|  | mouse mAB anti-GAPDH | MA5-15738 | 1:5000 |
|  | goat anti-mouse | 31430 | 1:5000 |
|  | Profilin 1 polyclonal Antibody | PA5-17444 | 1:1000 |
| R&D systems | mouse monoclonal anti-testican-1/SPOCK1 | MAB2327 | 1:500 |
