# Supplementary material for: A protein complex of LCN2, LOXL2 and MMP9 facilitates tumour metastasis in oesophageal cancer
Source: Mol Oncol. 2023 Oct 4;17(11):2451–71. doi: 10.1002/1878-0261.13529 (PMC10620126; doi:10.1002/1878-0261.13529)
Supplement: Supplementary file 12 — Table S2. Peptides used to Identify LCN2 Protein by MS. [file MOL2-17-2451-s009.docx]

| **Supplementary Table 2. Peptides used to Identify LCN2 Protein by MS** | | | | |
| --- | --- | --- | --- | --- |
| **No.** | **Sequence** | **PEP** | **Charge** | **Molecular Weight (Da)** |
| 1 | VPLQQNFQDNQFQGK | 9.224E-11 | 2 | 1790.88 |
| 2 | MYATIYELKEDK | 2.411E-08 | 3 | 1503.74 |
| 3 | SYPGLTSYLVR | 0.0004464 | 2 | 1255.66 |
| 4 | VVSTNYNQHAMVFFKK | 0.00118 | 4 | 1928.96 |
| 5 | ELTSELKENFIR | 0.000009 | 3 | 1478.78 |
| PEP: value represents the reliability of mass spectrometric identification of peptide segments, usually the standard is 0.1 or 0.5, and the lower the value, the higher the reliability of peptide segment identification. | | | | |
